# Racial disparity in curative treatment and survival from solid-organ cancers

**DOI:** 10.1093/bjs/znab089

**Published:** 2021-04-06

**Authors:** S K Kamarajah, P Sylla, S R Markar

**Affiliations:** 1 Department of Surgery, Queen Elizabeth Hospital Birmingham, University Hospital Birmingham NHS Trust, Birmingham, UK; 2 Northern Oesophagogastric Unit, Royal Victoria Infirmary, Newcastle University Trust Hospitals, Newcastle upon Tyne, UK; 3 Institute of Cancer and Genomic Sciences, College of Medical and Dental Sciences, University of Birmingham, Birmingham, UK; 4 Department of Surgery, Icahn School of Medicine at Mount Sinai, New York, New York, USA; 5 Department of Surgery and Cancer, Imperial College London, London, UK; 6 Department of Molecular Medicine and Surgery, Karolinska Institutet, Stockholm, Sweden

## Abstract

Race is an important prognostic factor affecting allocation to intervention and survival from cancer treatment in the USA. The mechanism of this association is an important area for future investigation.

## Introduction

Disparities in cancer care provision often lead to wide heterogeneity in oncological outcomes, which can affect patient outcomes negatively in more vulnerable populations. However, recognizing and understanding factors that influence disparities in cancer care is a necessary first step before intervening to eliminate these disparities. The impact of disparity on receipt of curative cancer treatment and long-term oncological effects remains relatively unknown. Previous small studies have focused on the role of race on survival in single-solid organ malignancy, and failed to explore these associations with outcomes in a broader context^1^^,^^2^. Furthermore, previous studies grouped all ethnic minorities together, and therefore lack granularity to identify disparities by individual ethnic minorities (black, Hispanic, Asian, other). As race is often closely associated with other risk factors for unequal access to specialized cancer treatment, such as socioeconomic status, education level, and insurance status^3^^,^^4^, any investigation must be performed using data sets that allow accurate adjustment for these factors in the analysis. Therefore, it is imperative to understand the pattern of treatment allocation by race with appropriate confounding factor adjustments.

Using the National Cancer Database (NCDB) from the USA, this study aimed to characterize the impact of race on allocation of curative surgery and neoadjuvant therapy, and long-term survival in patients with non-metastatic cancer across eight common cancers including those of the oesophagus, stomach, liver, pancreas, colon, rectum, breast, and lung.

## Methods

The NCDB, a joint project of the Commission on Cancer of the American College of Surgeons and the American Cancer Society^5^^,^^6^, was used to identify patients diagnosed with a non-metastatic solid-organ cancer (oesophageal, gastric, liver, pancreatic, colonic, rectal, breast, and lung) according to the ICD-O-3 codes from 2004 to 2016. Exclusion criteria were: metastatic cancer at diagnosis, other concurrent cancer diagnoses, and palliative treatment. Details of data collected and statistical analysis are available in *Appendix S1.*

## Results

This study included 3 718 367 patients with non-metastatic oesophageal (102 033), gastric (95 533), liver (149 715), pancreatic (173 296), colonic (805 204), rectal (114 649), breast (1 314 521), and lung (963 416) tumours. Black race was associated with a greater prevalence of medical co-morbidities, lower median income, advanced tumour stage (T3 or T4) (*Fig. S1a*), and node-positive disease (*Fig. S1b*) across seven of eight cancers studied (*Tables S1–S8*).

### Receipt of curative surgery

Receipt of curative surgery varied from 11.6 to 94.5 per cent for white patients compared with 8.3–93.0 per cent for black race across these cancers (*Table 1*). In adjusted analyses, black race was independently associated with significantly lower rates of receipt of surgery compared with white patients for seven of eight cancers studied: oesophageal (odds ratio (OR) 0.29, 95 per cent c.i. 0.28 to 0.31), liver (OR 0.76, 0.74 to 0.79), pancreatic (OR 0.72, 0.70 to 0.75), colonic (OR 0.76, 0.74 to 0.78), rectal (OR 0.67, 0.63 to 0.70), breast (OR 0.55, 0.54 to 0.56), and lung (OR 0.71, 0.70 to 0.72). Similar results were seen for Asian and Hispanic race (*Table 1*). Physician recommendation was the most common reason for patients not having surgery across all cancers (*Table S9*).

**Table 1:** Impact of race on receipt to curative cancer surgery and neoadjuvant therapy in non-metastatic cancers

	Receipt of surgery				Receipt of neoadjuvant therapy			
	No. of patients	Odds ratio	No. of patients	Odds ratio	No. of patients	Odds ratio	No. of patients	Odds ratio
	Oesophageal		Stomach		Oesophageal		Stomach	
White	37 396 (43.8)	1.00 (reference)	39631 (60.4)	1.00 (reference)	20 033 (23.4)	1.00 (reference)	12488 (19.0)	1.00 (reference)
Black	1796 (18.6)	0.29 (0.28, 0.31) *P* < 0.001	8196 (61.5)	1.05 (1.01, 1.09) *P* = 0.015	977 (10.1)	0.37 (0.34, 0.39) *P* < 0.001	1390 (10.4)	0.50 (0.47, 0.53) *P* < 0.001
Hispanic	1120 (33.0)	0.63 (0.59, 0.68) *P* < 0.001	5355 (64.8)	1.21 (1.15, 1.27) *P* < 0.001	612 (18.0)	0.72 (0.66, 0.79) *P* < 0.001	1172 (14.2)	0.70 (0.66, 0.75) *P* < 0.001
Asian	532 (30.7)	0.57 (0.51, 0.63) *P* < 0.001	4528 (72.5)	1.73 (1.63, 1.83) *P* < 0.001	316 (18.3)	0.73 (0.64, 0.82) *P* < 0.001	675 (10.8)	0.52 (0.47, 0.56) *P* < 0.001
Other	755 (41.6)	0.92 (0.83, 1.01) *P* = 0.069	1282 (60.6)	1.01 (0.92, 1.10) *P* = 0.856	331 (18.2)	0.73 (0.65, 0.82) *P* < 0.001	318 (15.0)	0.75 (0.67, 0.85) *P* < 0.001
	**Liver**		**Pancreas**		**Liver**		**Pancreas**	
White	28 947 (31.0)	1.00 (reference)	51970 (37.8)	1.00 (reference)	6912 (7.4)	1.00 (reference)	9591 (7.0)	1.00 (reference)
Black	5727 (25.4)	0.76 (0.74, 0.79) *P* < 0.001	6013 (30.6)	0.72 (0.70, 0.75) *P* < 0.001	1376 (6.1)	0.82 (0.77, 0.87) *P* < 0.001	1038 (5.3)	0.74 (0.69, 0.79) *P* < 0.001
Hispanic	4678 (25.5)	0.76 (0.74, 0.79) *P* < 0.001	2862 (34.6)	0.87 (0.83, 0.91) *P* < 0.001	1409 (7.7)	1.04 (0.98, 1.10) *P* = 0.189	422 (5.1)	0.72 (0.65, 0.79) *P* < 0.001
Asian	4183 (38.7)	1.41 (1.35, 1.47) *P* < 0.001	1454 (35.3)	0.90 (0.84, 0.96) *P* = 0.001	911 (8.4)	1.15 (1.07, 1.24) *P* < 0.001	231 (5.6)	0.79 (0.69, 0.90) *P* = 0.001
Other	1330 (29.6)	0.94 (0.88, 1.00) *P* = 0.051	1149 (30.9)	0.73 (0.68, 0.79) *P* < 0.001	358 (8.0)	1.08 (0.97, 1.21) *P* = 0.154	237 (6.4)	0.91 (0.79, 1.03) *P* = 0.150
	**Colon**		**Rectum**		**Colon**		**Rectum**	
White	602 391 (94.5)	1.00 (reference)	79 796 (84.0)	1.00 (reference)	16 603 (2.6)	1.00 (reference)	35 287 (37.2)	1.00 (reference)
Black	87 075 (93.0)	0.76 (0.74, 0.78) *P* < 0.001	8643 (77.8)	0.67 (0.63, 0.70) *P* < 0.001	2380 (2.5)	0.97 (0.93, 1.02) *P* = 0.242	3870 (34.8)	0.90 (0.87, 0.94) *P* < 0.001
Hispanic	37 021 (93.8)	0.87 (0.84, 0.91) *P* < 0.001	3511 (83.1)	0.93 (0.86, 1.01) *P* = 0.104	1317 (3.3)	1.29 (1.22, 1.37) *P* < 0.001	1563 (37.0)	0.99 (0.93, 1.06) *P* = 0.823
Asian	20 355 (94.2)	0.94 (0.89, 0.99) *P* = 0.031	2006 (84.1)	1.00 (0.90, 1.12) *P* = 0.943	785 (3.6)	1.41 (1.31, 1.51) *P* < 0.001	971 (40.7)	1.16 (1.07, 1.26) *P* < 0.001
Other	12 174 (91.5)	0.62 (0.58, 0.66) *P* < 0.001	1507 (77.2)	0.64 (0.58, 0.72) *P* < 0.001	494 (3.7)	1.44 (1.31, 1.58) *P* < 0.001	601 (30.8)	0.75 (0.68, 0.83) *P* < 0.001
	**Breast**		**Lung**		**Breast**		**Lung**	
White	975 958 (92.9)	1.00 (reference)	299649 (36.8)	1.00 (reference)	135 520 (7.9)	1.00 (reference)	25 664 (3.1)	1.00 (reference)
Black	121 942 (87.8)	0.55 (0.54, 0.56) *P* < 0.001	27931 (29.3)	0.71 (0.70, 0.72) *P* < 0.001	30 384 (13.1)	1.77 (1.75, 1.79) *P* < 0.001	2723 (2.9)	0.91 (0.87, 0.94) *P* < 0.001
Hispanic	56 075 (88.5)	0.59 (0.57, 0.60) *P* < 0.001	8086 (34.7)	0.92 (0.89, 0.94) *P* < 0.001	14 322 (13.0)	1.74 (1.71, 1.78) *P* < 0.001	789 (3.4)	1.08 (1.00, 1.16) *P* = 0.038
Asian	31 992 (90.6)	0.74 (0.71, 0.76) *P* < 0.001	6598 (40.8)	1.19 (1.15, 1.23) *P* < 0.001	6568 (10.3)	1.35 (1.32, 1.39) *P* < 0.001	646 (4.0)	1.28 (1.18, 1.39) *P* < 0.001
Other	22 169 (84.4)	0.41 (0.40, 0.43) *P* < 0.001	4349 (32.7)	0.83 (0.80, 0.87) *P* < 0.001	4348 (10.3)	1.34 (1.30, 1.39) *P* < 0.001	565 (4.2)	1.36 (1.25, 1.48) *P* < 0.001

Values in parentheses are 95 per cent confidence intervals. Binary logistic regression models were adjusted for race, centre volume quintile, facility type, facility location, age at diagnosis, sex, Charlson–Deyo co-morbidity score, insurance status, education level, median income, residence, AJCC clinical T category, and AJCC clinical N category.

### Receipt of neoadjuvant therapy

Receipt of neoadjuvant therapy varied from 2.6 to 37.2 per cent for white patients compared with 2.5 –34.8 per cent for black race across these cancers (*Table 1*). In adjusted analyses, black race was independently associated with significantly lower rates of receipt of neoadjuvant therapy than in white patients for six of eight cancers studied (*Table 1*).

### Long-term survival

**Fig. 1:**
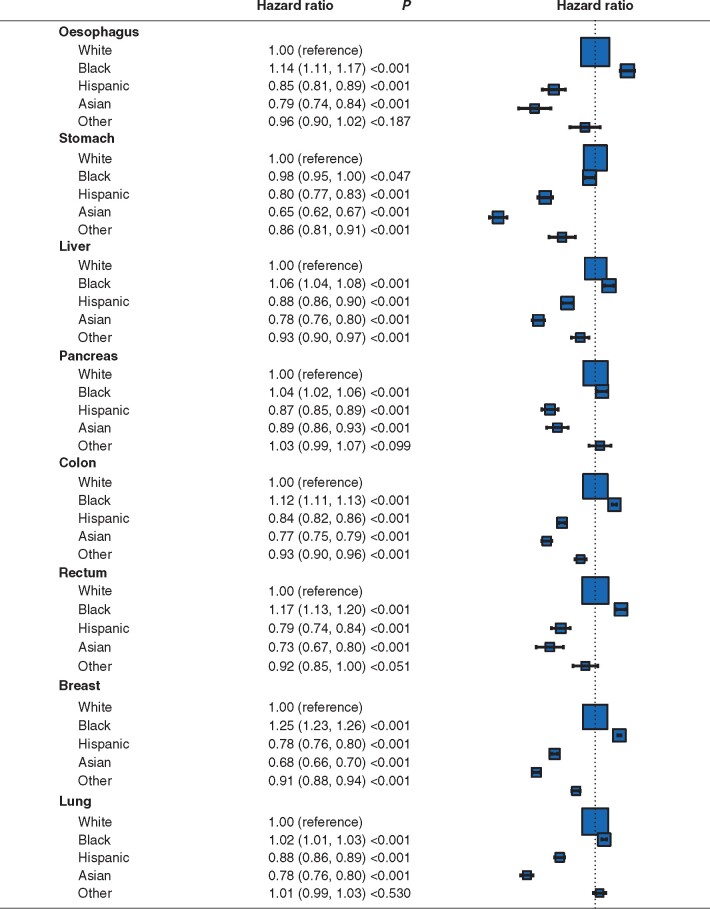
Adjusted Cox regression analyses of the impact of race on overall survival Hazard ratios for overall survival are shown with 95 per cent confidence intervals, for patients with cancer of the oesophagus, stomach, liver, pancreas, colon, rectum, breast, and lung. Cox regression models were adjusted for race, centre volume quintile, facility type, facility location, age at diagnosis, sex, Charlson–Deyo co-morbidity score, insurance status, education level, median income, residence, AJCC clinical T category, and AJCC clinical N category.

Median follow-up for the entire cohort was 25 (i.q.r. 19–36) months. Median survival for patients of black race was significantly shorter than that for white patients across oesophageal (12 *versus* 19 months), gastric (19 *versus* 20 months), liver (13 *versus* 14 months), pancreatic (10 *versus* 11 months), rectal (80 *versus* 93 months), and lung (20 *versus* 21 months) cancers (*Fig. S2*). Adjusted Cox regression analyses showed that black race was associated with significantly reduced survival for oesophageal (hazard ratio (HR) 1.14, 95 per cent c.i. 1.11 to 1.17), liver (HR 1.06, 1.04 to 1.08), pancreatic (HR 1.04, 1.02 to 1.06), colonic (HR 1.12, 1.11 to 1.13), rectal (1.17, 1.13 to 1.20), breast (HR 1.25, 1.23 to 1.26), and lung (HR 1.02, 1.01 to 1.03) cancers (*Fig. 1*). However, Asian or Hispanic race was often associated with significantly lower mortality rates than white race across all non-metastatic solid organ cancers (*Fig. 1*).

### Stratified analyses by time cohorts

Stratified analyses were performed by time cohorts (2004–2007, 2008–2011, 2012–2016), which demonstrated consistent results for receipt of surgery (*Table S10*), receipt of neoadjuvant therapy (*Table S11*), and reflect long-term survival than chemotherapy (*Table S12*).

## Discussion

This population-based cohort study of almost 4 million patients with non-metastatic cancer identified race as an important determinant of receipt of curative surgical resection and neoadjuvant therapy, and as a prognostic factor for long-term survival. Black race was associated with advanced T and N category at presentation, and was independently associated with lower rates of receipt of curative surgery, neoadjuvant therapy, and worse survival.

More advanced clinical presentation in ethnic minorities may be secondary to several factors^7^^,^^8^. First, poor attendance at screening programmes for specific cancers, and distrust in the healthcare system may result in lower rates of early cancer diagnosis^9^^,^^10^. However, discerning which factor is ultimately responsible and driving an interventional change is challenging. Second, a complex interaction between socioeconomic status and access to cancer care may influence delay in presentation of ethnic minorities^9^^,^^10^. This may be exacerbated further by COVID. Large initiatives are being rolled out to address these issues.

Previous descriptive, single-region studies have demonstrated lower rates of surgery^11–15^ and poor long-term survival^11^^,^^16^ in black race across different cancers. There may be several explanations for the lower rates of curative surgery and survival in the present study. First, this could be due to refusal to undergo surgical intervention, misunderstanding of treatment guidelines on the part of the treating physician, or contraindications to surgery among black race. Second, previous studies have shown that rates of surgery vary according to socioeconomic status, and it is hypothesized that this is due to communication and financial barriers^17^. Previous reports demonstrated that the likelihood of undergoing surgery increased when travelling more than 5 miles, which may be explained by travel associated with seeking tertiary-care centres^18^. This could adversely affect patients of low socioeconomic status who may be unable to travel for care. Finally, studies of hospital‐level variation in surgical practice have demonstrated that regional referral to high‐volume centres may have a positive effect on outcomes^19–22^.

Race is an important prognostic factor affecting receipt of surgical intervention and cancer survival in the USA. The findings of this study highlight the importance of implementing changes aimed at narrowing the disparities in outcomes between race in patients with cancer. Additional prospective analyses are warranted to further investigate the role of race in treatment decision-making and survival, and to identify specific hospital-level factors that affect disease management.

*Disclosure.* The authors declare no conflict of interest.

## Supplementary material

Supplementary material is available at *BJS* online

## Supplementary Material

znab089_Supplementary_Data
