# Supplementary material for: Racial disparity in curative treatment and survival from solid-organ cancers
Source: Br J Surg. 2021 Apr 6;108(9):1017–21. doi: 10.1093/bjs/znab089 (PMC10364912; doi:10.1093/bjs/znab089)
Supplement: znab089_Supplementary_Data [file znab089_supplementary_data.zip › R1_Appendix S1 v1_29Jan21_srm.docx]

*Data Source*

The National Cancer Data Base (NCDB) is a joint project of the Commission on Cancer (CoC) of the American College of Surgeons and the American Cancer Society.^5,6^ The NCDB gathers information from approximately 1,500 CoC-accredited hospitals and includes more than 70% of all newly diagnosed malignancies in the USA. It contains specific details about patient demographics (age, sex, race, insurance status, Charlson-Deyo comorbidities (CDCC)), facility type (i.e. community, academic, others) and location, tumour characteristics (size, grade, stage, histology), treatment course (type of surgery, receipt of chemotherapy, and radiation therapy), and outcomes (resection margins, lymph node status, 30-day readmission, 30-day and 90-day mortality and long-term survival).

*Study Population*

*Inclusion criteria*

Patients diagnosed with a non-metastatic solid organ cancer (i.e. oesophageal, gastric, liver, pancreas, colon, rectal, breast and lung) according to the International Classification of Disease for Oncology, Third Edition (ICD-O-3) from 2004 to 2016.

*Exclusion criteria*

The exclusion criteria were: (i) metastatic cancers at diagnosis, (ii) other concurrent cancer diagnoses, and (iii) patients receiving palliative treatment.

*Study Definitions*

We analysed the following patient-level characteristics as provided by NCDB: age (<60 vs. ≥60 years old), race (white, black, asian, hispanic, other), Charlson/Deyo comorbidity score,^7^ year of diagnosis, insurance status (Medicaid / Medicare, Private Insurance, Uninsured), zip code-level education status (<7%, 7% - 12.9%, 13% - 20.9%, ≥21%), zip code-level median household income (<$48,000, $48,000 - $62,999, ≥$63,000), and urban versus rural area of residence. The zip-code level education status represents the proportion of adults in the patient's zip code who did not graduate from high school and is categorized as equally proportioned quartiles among all US zip codes. The cut-offs for education status and household income were provided within the database. However, cut-offs for age were derived from the median of the database. We also analysed the following hospital-level characteristics: facility type (academic, community, other), facility location (Midwest, Northeast, South, West), and hospital volume (by cancers diagnosed per year;). Finally, we analysed the following clinicopathologic characteristics: clinical T status and N status (cN0, cN+, cNx).

*Statistical Analysis*

Categorical variables were compared using the chi-squared test. Non-normally distributed data were analysed using the Mann-Whitney U test. Survival was estimated using Kaplan-Meier survival curves and compared using the log-rank test. Multivariable analyses used binary logistic regression and Cox proportional hazards models adjusting for hospital-level (i.e. cancer centre volume quintile, facility type, facility location), patient-level (age at diagnosis, sex, CDCC score, insurance status, education level, median income, residence) and tumour-level (i.e. AJCC clinical T and clinical N stage) confounding factors. A p-value of <0.05 was considered to be statistically significant. Data analysis was performed using R Foundation Statistical software (R 3.2.2) with TableOne, ggplot2, Hmisc, Matchit and survival packages (R Foundation for Statistical Computing, Vienna, Austria).
