# Supplementary material for: Racial disparity in curative treatment and survival from solid-organ cancers
Source: Br J Surg. 2021 Apr 6;108(9):1017–21. doi: 10.1093/bjs/znab089 (PMC10364912; doi:10.1093/bjs/znab089)
Supplement: znab089_Supplementary_Data [file znab089_supplementary_data.zip › Supplementary tables and figures.docx]

Supplementary Table 1 Clinicopathologic characteristics of patients with non-metastatic oesophageal cancers by race

|  |  | White | Black | Hispanic | Asian | Other | p |
| --- | --- | --- | --- | --- | --- | --- | --- |
| Centre Volume Quintile | Quintile 1 | 17420 (20.4) | 1974 (20.5) | 740 (21.8) | 439 (25.4) | 252 (13.9) | <0.001 |
|  | Quintile 2 | 16716 (19.6) | 2091 (21.7) | 755 (22.3) | 290 (16.8) | 275 (15.2) |  |
|  | Quintile 3 | 16512 (19.3) | 2400 (24.9) | 833 (24.6) | 313 (18.1) | 261 (14.4) |  |
|  | Quintile 4 | 17123 (20.0) | 1874 (19.4) | 604 (17.8) | 410 (23.7) | 425 (23.4) |  |
|  | Quintile 5 | 17680 (20.7) | 1307 (13.5) | 459 (13.5) | 279 (16.1) | 601 (33.1) |  |
| Facility Type | Community | 39329 (46.0) | 3927 (40.7) | 1305 (38.5) | 683 (39.5) | 520 (28.7) | <0.001 |
|  | Academic | 34734 (40.6) | 4407 (45.7) | 1609 (47.4) | 863 (49.9) | 1057 (58.3) |  |
|  | Others | 11388 (13.3) | 1312 (13.6) | 477 (14.1) | 185 (10.7) | 237 (13.1) |  |
| Facility Location | Northeast | 19952 (23.3) | 1593 (16.5) | 786 (23.2) | 448 (25.9) | 418 (23.0) | <0.001 |
|  | South | 28232 (33.0) | 5635 (58.4) | 1203 (35.5) | 296 (17.1) | 437 (24.1) |  |
|  | Midwest | 24349 (28.5) | 1936 (20.1) | 315 (9.3) | 205 (11.8) | 441 (24.3) |  |
|  | West | 12918 (15.1) | 482 (5.0) | 1087 (32.1) | 782 (45.2) | 518 (28.6) |  |
| Age at Diagnosis, years | <55 | 10985 (12.9) | 1835 (19.0) | 654 (19.3) | 256 (14.8) | 297 (16.4) | <0.001 |
|  | 55-59 | 9611 (11.2) | 1601 (16.6) | 418 (12.3) | 192 (11.1) | 227 (12.5) |  |
|  | 60-64 | 12539 (14.7) | 1691 (17.5) | 487 (14.4) | 236 (13.6) | 262 (14.4) |  |
|  | 65-69 | 13762 (16.1) | 1498 (15.5) | 533 (15.7) | 244 (14.1) | 312 (17.2) |  |
|  | 70-74 | 12894 (15.1) | 1176 (12.2) | 490 (14.5) | 273 (15.8) | 262 (14.4) |  |
|  | 75-79 | 10894 (12.7) | 899 (9.3) | 325 (9.6) | 241 (13.9) | 190 (10.5) |  |
|  | 80-84 | 8166 (9.6) | 541 (5.6) | 278 (8.2) | 169 (9.8) | 162 (8.9) |  |
|  | 85+ | 6600 (7.7) | 405 (4.2) | 206 (6.1) | 120 (6.9) | 102 (5.6) |  |
| Sex | Male | 66629 (78.0) | 6372 (66.1) | 2683 (79.1) | 1260 (72.8) | 1392 (76.7) | <0.001 |
|  | Female | 18822 (22.0) | 3274 (33.9) | 708 (20.9) | 471 (27.2) | 422 (23.3) |  |
| CDCC Score | 0 | 60829 (71.2) | 6880 (71.3) | 2507 (73.9) | 1356 (78.3) | 1372 (75.6) | <0.001 |
|  | 1 | 17349 (20.3) | 1883 (19.5) | 639 (18.8) | 284 (16.4) | 319 (17.6) |  |
|  | 2 | 5089 (6.0) | 577 (6.0) | 144 (4.2) | 61 (3.5) | 83 (4.6) |  |
|  | 3 | 2184 (2.6) | 306 (3.2) | 101 (3.0) | 30 (1.7) | 40 (2.2) |  |
| Insurance Status | Uninsured | 1740 (2.0) | 692 (7.2) | 284 (8.4) | 116 (6.7) | 47 (2.6) | <0.001 |
|  | Private Insurance | 27840 (32.6) | 2116 (21.9) | 967 (28.5) | 560 (32.4) | 598 (33.0) |  |
|  | Medicaid | 3793 (4.4) | 1861 (19.3) | 446 (13.2) | 269 (15.5) | 134 (7.4) |  |
|  | Medicare | 48092 (56.3) | 4521 (46.9) | 1505 (44.4) | 741 (42.8) | 819 (45.1) |  |
|  | Other/Unknown | 3986 (4.7) | 456 (4.7) | 189 (5.6) | 45 (2.6) | 216 (11.9) |  |
| Education Level | >21% | 10376 (12.1) | 3876 (40.2) | 1650 (48.7) | 395 (22.8) | 279 (15.4) | <0.001 |
|  | 13%-20.9% | 21971 (25.7) | 3494 (36.2) | 798 (23.5) | 350 (20.2) | 454 (25.0) |  |
|  | 7%-12.9% | 31217 (36.5) | 1668 (17.3) | 628 (18.5) | 483 (27.9) | 648 (35.7) |  |
|  | <7% | 21497 (25.2) | 563 (5.8) | 297 (8.8) | 495 (28.6) | 420 (23.2) |  |
|  | Unknown | 390 (0.5) | 45 (0.5) | 18 (0.5) | 8 (0.5) | 13 (0.7) |  |
| Median Income | </=$47,999 | 33550 (39.3) | 6983 (72.4) | 1769 (52.2) | 411 (23.7) | 684 (37.7) | <0.001 |
|  | $48,000-$62,999 | 24120 (28.2) | 1551 (16.1) | 863 (25.4) | 445 (25.7) | 512 (28.2) |  |
|  | $63,000 + | 27324 (32.0) | 1058 (11.0) | 739 (21.8) | 867 (50.1) | 605 (33.4) |  |
|  | Unknown | 457 (0.5) | 54 (0.6) | 20 (0.6) | 8 (0.5) | 13 (0.7) |  |
| Residence | Metro | 69585 (81.4) | 8470 (87.8) | 3171 (93.5) | 1683 (97.2) | 1477 (81.4) | <0.001 |
|  | Urban/Rural | 15866 (18.6) | 1176 (12.2) | 220 (6.5) | 48 (2.8) | 337 (18.6) |  |
| Tumour Histology | Adenocarcinoma | 63323 (74.1) | 1410 (14.6) | 1836 (54.1) | 411 (23.7) | 1135 (62.6) | <0.001 |
|  | SCC | 22128 (25.9) | 8236 (85.4) | 1555 (45.9) | 1320 (76.3) | 679 (37.4) |  |
| AJCC Clinical T Stage | T0-1 | 15723 (18.4) | 1435 (14.9) | 528 (15.6) | 250 (14.4) | 394 (21.7) | <0.001 |
|  | T2 | 11132 (13.0) | 974 (10.1) | 411 (12.1) | 200 (11.6) | 190 (10.5) |  |
|  | T3-4 | 33863 (39.6) | 4164 (43.2) | 1307 (38.5) | 800 (46.2) | 638 (35.2) |  |
|  | Tx | 24733 (28.9) | 3073 (31.9) | 1145 (33.8) | 481 (27.8) | 592 (32.6) |  |
| AJCC Clinical N Stage | N0 | 36087 (42.2) | 3817 (39.6) | 1259 (37.1) | 640 (37.0) | 731 (40.3) | <0.001 |
|  | N+ | 30891 (36.2) | 3608 (37.4) | 1287 (38.0) | 760 (43.9) | 597 (32.9) |  |
|  | Nx | 18473 (21.6) | 2221 (23.0) | 845 (24.9) | 331 (19.1) | 486 (26.8) |  |

Supplementary Table 2 Clinicopathologic characteristics of patients with non-metastatic stomach cancers by race

|  |  | White | Black | Hispanic | Asian | Other | p-value |
| --- | --- | --- | --- | --- | --- | --- | --- |
| Centre Volume Quintile | Quintile 1 | 14683 (22.4) | 2457 (18.5) | 1122 (13.6) | 646 (10.3) | 303 (14.3) | <0.001 |
|  | Quintile 2 | 12986 (19.8) | 3039 (22.8) | 1626 (19.7) | 1039 (16.6) | 329 (15.6) |  |
|  | Quintile 3 | 13034 (19.9) | 2810 (21.1) | 1868 (22.6) | 1330 (21.3) | 357 (16.9) |  |
|  | Quintile 4 | 12620 (19.2) | 2743 (20.6) | 1926 (23.3) | 1303 (20.9) | 438 (20.7) |  |
|  | Quintile 5 | 12271 (18.7) | 2268 (17.0) | 1718 (20.8) | 1929 (30.9) | 688 (32.5) |  |
| Facility Type | Community | 31261 (47.7) | 5359 (40.2) | 3554 (43.0) | 2681 (42.9) | 692 (32.7) | <0.001 |
|  | Academic | 24633 (37.6) | 5730 (43.0) | 3373 (40.8) | 2790 (44.7) | 1097 (51.9) |  |
|  | Others | 9700 (14.8) | 2228 (16.7) | 1333 (16.1) | 776 (12.4) | 326 (15.4) |  |
| Facility Location | Northeast | 17436 (26.6) | 2530 (19.0) | 1715 (20.8) | 1567 (25.1) | 530 (25.1) | <0.001 |
|  | South | 20905 (31.9) | 7271 (54.6) | 2855 (34.6) | 1038 (16.6) | 526 (24.9) |  |
|  | Midwest | 17381 (26.5) | 2601 (19.5) | 674 (8.2) | 574 (9.2) | 411 (19.4) |  |
|  | West | 9872 (15.1) | 915 (6.9) | 3016 (36.5) | 3068 (49.1) | 648 (30.6) |  |
| Age at Diagnosis, years | <55 | 7331 (11.2) | 2115 (15.9) | 1898 (23.0) | 950 (15.2) | 353 (16.7) | <0.001 |
|  | 55-59 | 5454 (8.3) | 1373 (10.3) | 781 (9.5) | 565 (9.0) | 205 (9.7) |  |
|  | 60-64 | 7255 (11.1) | 1507 (11.3) | 882 (10.7) | 655 (10.5) | 265 (12.5) |  |
|  | 65-69 | 8996 (13.7) | 1699 (12.8) | 1042 (12.6) | 833 (13.3) | 320 (15.1) |  |
|  | 70-74 | 9394 (14.3) | 1847 (13.9) | 1135 (13.7) | 852 (13.6) | 272 (12.9) |  |
|  | 75-79 | 9681 (14.8) | 1768 (13.3) | 1047 (12.7) | 910 (14.6) | 291 (13.8) |  |
|  | 80-84 | 8644 (13.2) | 1455 (10.9) | 749 (9.1) | 783 (12.5) | 216 (10.2) |  |
|  | 85+ | 8839 (13.5) | 1553 (11.7) | 726 (8.8) | 699 (11.2) | 193 (9.1) |  |
| Sex | Male | 45799 (69.8) | 7912 (59.4) | 5152 (62.4) | 3855 (61.7) | 1396 (66.0) | <0.001 |
|  | Female | 19795 (30.2) | 5405 (40.6) | 3108 (37.6) | 2392 (38.3) | 719 (34.0) |  |
| CDCC Score | 0 | 43083 (65.7) | 8192 (61.5) | 5698 (69.0) | 4561 (73.0) | 1486 (70.3) | <0.001 |
|  | 1 | 15094 (23.0) | 3205 (24.1) | 1797 (21.8) | 1232 (19.7) | 446 (21.1) |  |
|  | 2 | 5095 (7.8) | 1257 (9.4) | 518 (6.3) | 321 (5.1) | 123 (5.8) |  |
|  | 3 | 2322 (3.5) | 663 (5.0) | 247 (3.0) | 133 (2.1) | 60 (2.8) |  |
| Insurance Status | Uninsured | 1013 (1.5) | 627 (4.7) | 851 (10.3) | 256 (4.1) | 71 (3.4) | <0.001 |
|  | Private Insurance | 19089 (29.1) | 3477 (26.1) | 2341 (28.3) | 1887 (30.2) | 654 (30.9) |  |
|  | Medicaid | 2299 (3.5) | 1192 (9.0) | 1168 (14.1) | 807 (12.9) | 169 (8.0) |  |
|  | Medicare | 40951 (62.4) | 7492 (56.3) | 3573 (43.3) | 3141 (50.3) | 990 (46.8) |  |
|  | Other/Unknown | 2242 (3.4) | 529 (4.0) | 327 (4.0) | 156 (2.5) | 231 (10.9) |  |
| Education Level | >21% | 8724 (13.3) | 4894 (36.8) | 4220 (51.1) | 1547 (24.8) | 444 (21.0) | <0.001 |
|  | 13%-20.9% | 16634 (25.4) | 4812 (36.1) | 1831 (22.2) | 1264 (20.2) | 507 (24.0) |  |
|  | 7%-12.9% | 23654 (36.1) | 2594 (19.5) | 1468 (17.8) | 1801 (28.8) | 675 (31.9) |  |
|  | <7% | 16285 (24.8) | 958 (7.2) | 704 (8.5) | 1606 (25.7) | 477 (22.6) |  |
|  | Unknown | 297 (0.5) | 59 (0.4) | 37 (0.4) | 29 (0.5) | 12 (0.6) |  |
| Median Income | </=$47,999 | 24741 (37.7) | 8944 (67.2) | 4143 (50.2) | 1485 (23.8) | 835 (39.5) | <0.001 |
|  | $48,000-$62,999 | 18496 (28.2) | 2437 (18.3) | 2210 (26.8) | 1591 (25.5) | 520 (24.6) |  |
|  | $63,000 + | 22027 (33.6) | 1874 (14.1) | 1866 (22.6) | 3142 (50.3) | 747 (35.3) |  |
|  | Unknown | 330 (0.5) | 62 (0.5) | 41 (0.5) | 29 (0.5) | 13 (0.6) |  |
| Residence | Metro | 54777 (83.5) | 12085 (90.7) | 7878 (95.4) | 6128 (98.1) | 1802 (85.2) | <0.001 |
|  | Urban/Rural | 10817 (16.5) | 1232 (9.3) | 382 (4.6) | 119 (1.9) | 313 (14.8) |  |
| AJCC Clinical T Stage | T0-1 | 11408 (17.4) | 2189 (16.4) | 1291 (15.6) | 1143 (18.3) | 364 (17.2) | <0.001 |
|  | T2 | 7508 (11.4) | 1178 (8.8) | 777 (9.4) | 623 (10.0) | 189 (8.9) |  |
|  | T3-4 | 18467 (28.2) | 2916 (21.9) | 2083 (25.2) | 1271 (20.3) | 514 (24.3) |  |
|  | Tx | 28211 (43.0) | 7034 (52.8) | 4109 (49.7) | 3210 (51.4) | 1048 (49.6) |  |
| AJCC Clinical N Stage | N0 | 28003 (42.7) | 5347 (40.2) | 3228 (39.1) | 2791 (44.7) | 801 (37.9) | <0.001 |
|  | N+ | 16345 (24.9) | 2713 (20.4) | 1916 (23.2) | 1156 (18.5) | 500 (23.6) |  |
|  | Nx | 21246 (32.4) | 5257 (39.5) | 3116 (37.7) | 2300 (36.8) | 814 (38.5) |  |

Supplementary Table 3 Clinicopathologic characteristics of patients with non-metastatic liver cancers by race

|  |  | White | Black | Hispanic | Asian | Other | p-value |
| --- | --- | --- | --- | --- | --- | --- | --- |
| Centre Volume Quintile | Quintile 1 | 21190 (22.7) | 4220 (18.7) | 2399 (13.1) | 1594 (14.8) | 670 (14.9) | <0.001 |
|  | Quintile 2 | 18182 (19.4) | 4773 (21.2) | 3929 (21.4) | 2130 (19.7) | 803 (17.9) |  |
|  | Quintile 3 | 17260 (18.5) | 4489 (19.9) | 5002 (27.2) | 2785 (25.8) | 1023 (22.7) |  |
|  | Quintile 4 | 18681 (20.0) | 5047 (22.4) | 3791 (20.6) | 1714 (15.9) | 918 (20.4) |  |
|  | Quintile 5 | 18209 (19.5) | 3988 (17.7) | 3252 (17.7) | 2583 (23.9) | 1083 (24.1) |  |
| Facility Type | Community | 28901 (30.9) | 5146 (22.9) | 5381 (29.3) | 3063 (28.3) | 1108 (24.6) | <0.001 |
|  | Academic | 51321 (54.9) | 14037 (62.3) | 10846 (59.0) | 6359 (58.8) | 2810 (62.5) |  |
|  | Others | 13300 (14.2) | 3334 (14.8) | 2146 (11.7) | 1384 (12.8) | 579 (12.9) |  |
| Facility Location | Northeast | 19777 (21.1) | 4475 (19.9) | 3635 (19.8) | 2343 (21.7) | 1099 (24.4) | <0.001 |
|  | South | 35722 (38.2) | 11240 (49.9) | 6950 (37.8) | 2152 (19.9) | 1226 (27.3) |  |
|  | Midwest | 21596 (23.1) | 4720 (21.0) | 1457 (7.9) | 1051 (9.7) | 762 (16.9) |  |
|  | West | 15534 (16.6) | 1626 (7.2) | 6075 (33.1) | 4932 (45.6) | 1315 (29.2) |  |
|  | Unknown | 893 (1.0) | 456 (2.0) | 256 (1.4) | 328 (3.0) | 95 (2.1) |  |
| Age at Diagnosis, years | <55 | 16377 (17.5) | 5003 (22.2) | 4411 (24.0) | 2441 (22.6) | 1036 (23.0) | <0.001 |
|  | 55-59 | 17349 (18.6) | 5396 (24.0) | 3494 (19.0) | 1567 (14.5) | 899 (20.0) |  |
|  | 60-64 | 16490 (17.6) | 5367 (23.8) | 3009 (16.4) | 1582 (14.6) | 804 (17.9) |  |
|  | 65-69 | 13047 (14.0) | 3214 (14.3) | 2671 (14.5) | 1603 (14.8) | 636 (14.1) |  |
|  | 70-74 | 10723 (11.5) | 1702 (7.6) | 1925 (10.5) | 1385 (12.8) | 480 (10.7) |  |
|  | 75-79 | 9153 (9.8) | 977 (4.3) | 1481 (8.1) | 1119 (10.4) | 366 (8.1) |  |
|  | 80-84 | 6571 (7.0) | 521 (2.3) | 874 (4.8) | 696 (6.4) | 178 (4.0) |  |
|  | 85+ | 3812 (4.1) | 337 (1.5) | 508 (2.8) | 413 (3.8) | 98 (2.2) |  |
| Sex | Male | 71889 (76.9) | 16738 (74.3) | 13514 (73.6) | 7765 (71.9) | 3342 (74.3) | <0.001 |
|  | Female | 21633 (23.1) | 5779 (25.7) | 4859 (26.4) | 3041 (28.1) | 1155 (25.7) |  |
| CDCC Score | 0 | 51200 (54.7) | 12857 (57.1) | 9801 (53.3) | 7369 (68.2) | 2679 (59.6) | <0.001 |
|  | 1 | 20483 (21.9) | 4952 (22.0) | 4048 (22.0) | 1922 (17.8) | 901 (20.0) |  |
|  | 2 | 7212 (7.7) | 1609 (7.1) | 1211 (6.6) | 460 (4.3) | 260 (5.8) |  |
|  | 3 | 14627 (15.6) | 3099 (13.8) | 3313 (18.0) | 1055 (9.8) | 657 (14.6) |  |
| Insurance Status | Uninsured | 3617 (3.9) | 1679 (7.5) | 1682 (9.2) | 637 (5.9) | 233 (5.2) | <0.001 |
|  | Private Insurance | 30511 (32.6) | 6243 (27.7) | 4860 (26.5) | 3961 (36.7) | 1452 (32.3) |  |
|  | Medicaid | 9633 (10.3) | 4982 (22.1) | 3687 (20.1) | 1743 (16.1) | 759 (16.9) |  |
|  | Medicare | 46216 (49.4) | 8475 (37.6) | 7439 (40.5) | 4203 (38.9) | 1640 (36.5) |  |
|  | Other/Unknown | 3545 (3.8) | 1138 (5.1) | 705 (3.8) | 262 (2.4) | 413 (9.2) |  |
| Education Level | >21% | 15195 (16.2) | 8455 (37.5) | 10135 (55.2) | 3205 (29.7) | 1049 (23.3) | <0.001 |
|  | 13%-20.9% | 25862 (27.7) | 8065 (35.8) | 4087 (22.2) | 2375 (22.0) | 1214 (27.0) |  |
|  | 7%-12.9% | 31553 (33.7) | 4266 (18.9) | 2794 (15.2) | 2974 (27.5) | 1348 (30.0) |  |
|  | <7% | 20548 (22.0) | 1658 (7.4) | 1272 (6.9) | 2214 (20.5) | 856 (19.0) |  |
|  | Unknown | 364 (0.4) | 73 (0.3) | 85 (0.5) | 38 (0.4) | 30 (0.7) |  |
| Median Income | </=$47,999 | 39242 (42.0) | 15479 (68.7) | 10725 (58.4) | 3023 (28.0) | 1976 (43.9) | <0.001 |
|  | $48,000-$62,999 | 26091 (27.9) | 3964 (17.6) | 4414 (24.0) | 3014 (27.9) | 1115 (24.8) |  |
|  | $63,000 + | 27769 (29.7) | 2973 (13.2) | 3141 (17.1) | 4729 (43.8) | 1375 (30.6) |  |
|  | Unknown | 420 (0.4) | 101 (0.4) | 93 (0.5) | 40 (0.4) | 31 (0.7) |  |
| Residence | Metro | 78040 (83.4) | 21120 (93.8) | 17221 (93.7) | 10601 (98.1) | 3768 (83.8) | <0.001 |
|  | Urban/Rural | 15482 (16.6) | 1397 (6.2) | 1152 (6.3) | 205 (1.9) | 729 (16.2) |  |
| AJCC Clinical T Stage | T0-1 | 32239 (34.5) | 7231 (32.1) | 6433 (35.0) | 3763 (34.8) | 1494 (33.2) | <0.001 |
|  | T2 | 19558 (20.9) | 4359 (19.4) | 4070 (22.2) | 1992 (18.4) | 933 (20.7) |  |
|  | T3-4 | 20959 (22.4) | 6030 (26.8) | 3885 (21.1) | 2592 (24.0) | 1000 (22.2) |  |
|  | Tx | 20766 (22.2) | 4897 (21.7) | 3985 (21.7) | 2459 (22.8) | 1070 (23.8) |  |
| AJCC Clinical N Stage | N0 | 68976 (73.8) | 16505 (73.3) | 13678 (74.4) | 8051 (74.5) | 3217 (71.5) | <0.001 |
|  | N+ | 4728 (5.1) | 1309 (5.8) | 723 (3.9) | 353 (3.3) | 227 (5.0) |  |
|  | Nx | 19818 (21.2) | 4703 (20.9) | 3972 (21.6) | 2402 (22.2) | 1053 (23.4) |  |

Supplementary Table 4 Clinicopathologic characteristics of patients with non-metastatic pancreatic cancers by race

|  |  | White | Black | Hispanic | Asian | Other | p-value |
| --- | --- | --- | --- | --- | --- | --- | --- |
| Centre Volume Quintile | Quintile 1 | 28507 (20.7) | 3622 (18.4) | 1823 (22.0) | 886 (21.5) | 514 (13.8) | <0.001 |
|  | Quintile 2 | 26424 (19.2) | 4374 (22.2) | 1909 (23.1) | 933 (22.7) | 485 (13.0) |  |
|  | Quintile 3 | 26778 (19.5) | 4435 (22.5) | 1807 (21.8) | 837 (20.3) | 736 (19.8) |  |
|  | Quintile 4 | 28392 (20.6) | 3665 (18.6) | 1620 (19.6) | 572 (13.9) | 831 (22.3) |  |
|  | Quintile 5 | 27393 (19.9) | 3586 (18.2) | 1120 (13.5) | 890 (21.6) | 1156 (31.1) |  |
| Facility Type | Community | 52889 (38.5) | 5941 (30.2) | 3014 (36.4) | 1598 (38.8) | 940 (25.3) | <0.001 |
|  | Academic | 65925 (47.9) | 10800 (54.9) | 4235 (51.2) | 2156 (52.4) | 2311 (62.1) |  |
|  | Others | 18680 (13.6) | 2941 (14.9) | 1030 (12.4) | 364 (8.8) | 471 (12.7) |  |
| Facility Location | Northeast | 30780 (22.4) | 3248 (16.5) | 1485 (17.9) | 763 (18.5) | 837 (22.5) | <0.001 |
|  | South | 48136 (35.0) | 10954 (55.7) | 3211 (38.8) | 704 (17.1) | 1045 (28.1) |  |
|  | Midwest | 37161 (27.0) | 4198 (21.3) | 682 (8.2) | 421 (10.2) | 793 (21.3) |  |
|  | West | 21417 (15.6) | 1282 (6.5) | 2901 (35.0) | 2230 (54.2) | 1047 (28.1) |  |
| Age at Diagnosis, years | <55 | 14290 (10.4) | 3154 (16.0) | 1419 (17.1) | 514 (12.5) | 496 (13.3) | <0.001 |
|  | 55-59 | 13242 (9.6) | 2601 (13.2) | 891 (10.8) | 391 (9.5) | 391 (10.5) |  |
|  | 60-64 | 18026 (13.1) | 2987 (15.2) | 1119 (13.5) | 531 (12.9) | 500 (13.4) |  |
|  | 65-69 | 21431 (15.6) | 3040 (15.4) | 1245 (15.0) | 570 (13.8) | 577 (15.5) |  |
|  | 70-74 | 21717 (15.8) | 2833 (14.4) | 1264 (15.3) | 632 (15.3) | 551 (14.8) |  |
|  | 75-79 | 20615 (15.0) | 2391 (12.1) | 1114 (13.5) | 645 (15.7) | 555 (14.9) |  |
|  | 80-84 | 16365 (11.9) | 1607 (8.2) | 768 (9.3) | 487 (11.8) | 381 (10.2) |  |
|  | 85+ | 11808 (8.6) | 1069 (5.4) | 459 (5.5) | 348 (8.5) | 271 (7.3) |  |
| Sex | Male | 69773 (50.7) | 8599 (43.7) | 4096 (49.5) | 1907 (46.3) | 1884 (50.6) | <0.001 |
|  | Female | 67721 (49.3) | 11083 (56.3) | 4183 (50.5) | 2211 (53.7) | 1838 (49.4) |  |
| CDCC Score | 0 | 92459 (67.2) | 11988 (60.9) | 5319 (64.2) | 2929 (71.1) | 2697 (72.5) | <0.001 |
|  | 1 | 32635 (23.7) | 5546 (28.2) | 2284 (27.6) | 983 (23.9) | 742 (19.9) |  |
|  | 2 | 8613 (6.3) | 1383 (7.0) | 438 (5.3) | 146 (3.5) | 193 (5.2) |  |
|  | 3 | 3787 (2.8) | 765 (3.9) | 238 (2.9) | 60 (1.5) | 90 (2.4) |  |
| Insurance Status | Uninsured | 2586 (1.9) | 847 (4.3) | 670 (8.1) | 178 (4.3) | 135 (3.6) | <0.001 |
|  | Private Insurance | 42768 (31.1) | 5765 (29.3) | 2458 (29.7) | 1421 (34.5) | 1220 (32.8) |  |
|  | Medicaid | 4558 (3.3) | 1958 (9.9) | 1050 (12.7) | 365 (8.9) | 192 (5.2) |  |
|  | Medicare | 82551 (60.0) | 10275 (52.2) | 3730 (45.1) | 2043 (49.6) | 1872 (50.3) |  |
|  | Other/Unknown | 5031 (3.7) | 837 (4.3) | 371 (4.5) | 111 (2.7) | 303 (8.1) |  |
| Education Level | >21% | 16790 (12.2) | 6662 (33.8) | 3965 (47.9) | 861 (20.9) | 600 (16.1) | <0.001 |
|  | 13%-20.9% | 34006 (24.7) | 7033 (35.7) | 1872 (22.6) | 857 (20.8) | 913 (24.5) |  |
|  | 7%-12.9% | 48554 (35.3) | 4152 (21.1) | 1488 (18.0) | 1227 (29.8) | 1241 (33.3) |  |
|  | <7% | 37568 (27.3) | 1771 (9.0) | 916 (11.1) | 1155 (28.0) | 939 (25.2) |  |
|  | Unknown | 576 (0.4) | 64 (0.3) | 38 (0.5) | 18 (0.4) | 29 (0.8) |  |
| Median Income | </=$47,999 | 51867 (37.7) | 12784 (65.0) | 4120 (49.8) | 841 (20.4) | 1311 (35.2) | <0.001 |
|  | $48,000-$62,999 | 38297 (27.9) | 3739 (19.0) | 2169 (26.2) | 1029 (25.0) | 1017 (27.3) |  |
|  | $63,000 + | 46687 (34.0) | 3083 (15.7) | 1952 (23.6) | 2229 (54.1) | 1363 (36.6) |  |
|  | Unknown | 643 (0.5) | 76 (0.4) | 38 (0.5) | 19 (0.5) | 31 (0.8) |  |
| Residence | Metro | 112110 (81.5) | 17724 (90.1) | 7765 (93.8) | 3993 (97.0) | 3082 (82.8) | <0.001 |
|  | Urban/Rural | 25384 (18.5) | 1958 (9.9) | 514 (6.2) | 125 (3.0) | 640 (17.2) |  |
| AJCC Clinical T Stage | T0-1 | 8630 (6.3) | 990 (5.0) | 419 (5.1) | 264 (6.4) | 186 (5.0) | <0.001 |
|  | T2 | 26060 (19.0) | 3470 (17.6) | 1402 (16.9) | 686 (16.7) | 613 (16.5) |  |
|  | T3-4 | 62867 (45.7) | 9639 (49.0) | 3932 (47.5) | 2014 (48.9) | 1607 (43.2) |  |
|  | Tx | 39937 (29.0) | 5583 (28.4) | 2526 (30.5) | 1154 (28.0) | 1316 (35.4) |  |
| AJCC Clinical N Stage | N0 | 69912 (50.8) | 9954 (50.6) | 4089 (49.4) | 2206 (53.6) | 1719 (46.2) | <0.001 |
|  | N+ | 28492 (20.7) | 4294 (21.8) | 1720 (20.8) | 797 (19.4) | 687 (18.5) |  |
|  | Nx | 39090 (28.4) | 5434 (27.6) | 2470 (29.8) | 1115 (27.1) | 1316 (35.4) |  |

Supplementary Table 5 Clinicopathologic characteristics of patients with non-metastatic colon cancers by race

|  |  | White | Black | Hispanic | Asian | Other | p-value |
| --- | --- | --- | --- | --- | --- | --- | --- |
| Centre Volume Quintile | Quintile 1 | 132524 (20.8) | 16864 (18.0) | 7412 (18.8) | 3543 (16.4) | 2034 (15.3) | <0.001 |
|  | Quintile 2 | 127413 (20.0) | 17779 (19.0) | 7587 (19.2) | 4684 (21.7) | 2497 (18.8) |  |
|  | Quintile 3 | 128657 (20.2) | 18760 (20.0) | 6923 (17.5) | 4803 (22.2) | 2535 (19.1) |  |
|  | Quintile 4 | 127702 (20.0) | 18489 (19.7) | 7088 (18.0) | 4565 (21.1) | 2742 (20.6) |  |
|  | Quintile 5 | 120846 (19.0) | 21784 (23.3) | 10463 (26.5) | 4012 (18.6) | 3497 (26.3) |  |
| Facility Type | Community | 384745 (60.4) | 42693 (45.6) | 19369 (49.1) | 11086 (51.3) | 6009 (45.2) | <0.001 |
|  | Academic | 149349 (23.4) | 34330 (36.6) | 12405 (31.4) | 7483 (34.6) | 4666 (35.1) |  |
|  | Others | 103048 (16.2) | 16653 (17.8) | 7699 (19.5) | 3038 (14.1) | 2630 (19.8) |  |
| Facility Location | Northeast | 143587 (22.5) | 15738 (16.8) | 7614 (19.3) | 4473 (20.7) | 3302 (24.8) | <0.001 |
|  | South | 217165 (34.1) | 51403 (54.9) | 15643 (39.6) | 3628 (16.8) | 3528 (26.5) |  |
|  | Midwest | 179238 (28.1) | 19470 (20.8) | 3153 (8.0) | 2347 (10.9) | 3053 (22.9) |  |
|  | West | 97152 (15.2) | 7065 (7.5) | 13063 (33.1) | 11159 (51.6) | 3422 (25.7) |  |
| Age at Diagnosis, years | <55 | 94115 (14.8) | 21655 (23.1) | 10362 (26.3) | 4824 (22.3) | 3096 (23.3) | <0.001 |
|  | 55-59 | 53458 (8.4) | 11825 (12.6) | 4518 (11.4) | 2398 (11.1) | 1504 (11.3) |  |
|  | 60-64 | 65088 (10.2) | 12597 (13.4) | 4823 (12.2) | 2645 (12.2) | 1671 (12.6) |  |
|  | 65-69 | 80758 (12.7) | 12979 (13.9) | 5122 (13.0) | 2939 (13.6) | 1749 (13.1) |  |
|  | 70-74 | 85132 (13.4) | 11341 (12.1) | 4616 (11.7) | 2731 (12.6) | 1664 (12.5) |  |
|  | 75-79 | 90915 (14.3) | 9727 (10.4) | 4210 (10.7) | 2506 (11.6) | 1417 (10.7) |  |
|  | 80-84 | 84565 (13.3) | 7397 (7.9) | 3234 (8.2) | 1918 (8.9) | 1166 (8.8) |  |
|  | 85+ | 83111 (13.0) | 6155 (6.6) | 2588 (6.6) | 1646 (7.6) | 1038 (7.8) |  |
| Sex | Male | 315642 (49.5) | 43206 (46.1) | 20563 (52.1) | 10694 (49.5) | 6847 (51.5) | <0.001 |
|  | Female | 321500 (50.5) | 50470 (53.9) | 18910 (47.9) | 10913 (50.5) | 6458 (48.5) |  |
| CDCC Score | 0 | 433453 (68.0) | 62766 (67.0) | 27830 (70.5) | 16608 (76.9) | 9706 (73.0) | <0.001 |
|  | 1 | 139666 (21.9) | 21626 (23.1) | 8465 (21.4) | 3787 (17.5) | 2553 (19.2) |  |
|  | 2 | 43704 (6.9) | 6014 (6.4) | 2051 (5.2) | 814 (3.8) | 718 (5.4) |  |
|  | 3 | 20319 (3.2) | 3270 (3.5) | 1127 (2.9) | 398 (1.8) | 328 (2.5) |  |
| Insurance Status | Uninsured | 12041 (1.9) | 5113 (5.5) | 3405 (8.6) | 1041 (4.8) | 458 (3.4) | <0.001 |
|  | Private Insurance | 206232 (32.4) | 32200 (34.4) | 13683 (34.7) | 8838 (40.9) | 5143 (38.7) |  |
|  | Medicaid | 18080 (2.8) | 8422 (9.0) | 4816 (12.2) | 2296 (10.6) | 874 (6.6) |  |
|  | Medicare | 386398 (60.6) | 45108 (48.2) | 16449 (41.7) | 8948 (41.4) | 5908 (44.4) |  |
|  | Other/Unknown | 14391 (2.3) | 2833 (3.0) | 1120 (2.8) | 484 (2.2) | 922 (6.9) |  |
| Education Level | >21% | 81563 (12.8) | 32577 (34.8) | 19057 (48.3) | 4755 (22.0) | 2409 (18.1) | <0.001 |
|  | 13%-20.9% | 160287 (25.2) | 33676 (35.9) | 9163 (23.2) | 4474 (20.7) | 3191 (24.0) |  |
|  | 7%-12.9% | 226797 (35.6) | 19378 (20.7) | 7407 (18.8) | 6572 (30.4) | 4182 (31.4) |  |
|  | <7% | 165489 (26.0) | 7644 (8.2) | 3699 (9.4) | 5724 (26.5) | 3439 (25.8) |  |
|  | Unknown | 3006 (0.5) | 401 (0.4) | 147 (0.4) | 82 (0.4) | 84 (0.6) |  |
| Median Income | </=$47,999 | 241680 (37.9) | 61455 (65.6) | 19888 (50.4) | 4671 (21.6) | 4819 (36.2) | <0.001 |
|  | $48,000-$62,999 | 177181 (27.8) | 17327 (18.5) | 10544 (26.7) | 5581 (25.8) | 3440 (25.9) |  |
|  | $63,000 + | 214939 (33.7) | 14432 (15.4) | 8879 (22.5) | 11271 (52.2) | 4956 (37.2) |  |
|  | Unknown | 3342 (0.5) | 462 (0.5) | 162 (0.4) | 84 (0.4) | 90 (0.7) |  |
| Residence | Metro | 532189 (83.5) | 85441 (91.2) | 37670 (95.4) | 21184 (98.0) | 11376 (85.5) | <0.001 |
|  | Urban/Rural | 104953 (16.5) | 8235 (8.8) | 1803 (4.6) | 423 (2.0) | 1929 (14.5) |  |
| AJCC Clinical T Stage | T0-1 | 93499 (14.7) | 14668 (15.7) | 5655 (14.3) | 3248 (15.0) | 2131 (16.0) | <0.001 |
|  | T2 | 31087 (4.9) | 3956 (4.2) | 1761 (4.5) | 901 (4.2) | 506 (3.8) |  |
|  | T3-4 | 94612 (14.8) | 13932 (14.9) | 6577 (16.7) | 3410 (15.8) | 1983 (14.9) |  |
|  | Tx | 417944 (65.6) | 61120 (65.2) | 25480 (64.6) | 14048 (65.0) | 8685 (65.3) |  |
| AJCC Clinical N Stage | N0 | 314912 (49.4) | 44647 (47.7) | 19201 (48.6) | 11050 (51.1) | 6457 (48.5) | <0.001 |
|  | N+ | 47121 (7.4) | 7829 (8.4) | 3757 (9.5) | 2003 (9.3) | 1025 (7.7) |  |
|  | Nx | 275109 (43.2) | 41200 (44.0) | 16515 (41.8) | 8554 (39.6) | 5823 (43.8) |  |

Supplementary Table 6 Clinicopathologic characteristics of patients with non-metastatic rectal cancers by race

|  |  | White | Black | Hispanic | Asian | Other | p-value |
| --- | --- | --- | --- | --- | --- | --- | --- |
| Centre Volume Quintile | Quintile 1 | 19384 (20.4) | 2152 (19.4) | 734 (17.4) | 376 (15.8) | 293 (15.0) | <0.001 |
|  | Quintile 2 | 19407 (20.4) | 2272 (20.4) | 675 (16.0) | 368 (15.4) | 277 (14.2) |  |
|  | Quintile 3 | 19099 (20.1) | 2390 (21.5) | 675 (16.0) | 397 (16.6) | 365 (18.7) |  |
|  | Quintile 4 | 18224 (19.2) | 2279 (20.5) | 1333 (31.5) | 625 (26.2) | 445 (22.8) |  |
|  | Quintile 5 | 18859 (19.9) | 2019 (18.2) | 809 (19.1) | 620 (26.0) | 572 (29.3) |  |
| Facility Type | Community | 49139 (51.7) | 4515 (40.6) | 1685 (39.9) | 781 (32.7) | 624 (32.0) | <0.001 |
|  | Academic | 31570 (33.2) | 4748 (42.7) | 1792 (42.4) | 1252 (52.5) | 925 (47.4) |  |
|  | Others | 14264 (15.0) | 1849 (16.6) | 749 (17.7) | 353 (14.8) | 403 (20.6) |  |
| Facility Location | Northeast | 33773 (35.6) | 2609 (23.5) | 1926 (45.6) | 1192 (50.0) | 877 (44.9) | <0.001 |
|  | South | 31214 (32.9) | 5689 (51.2) | 1518 (35.9) | 686 (28.8) | 520 (26.6) |  |
|  | Midwest | 29986 (31.6) | 2814 (25.3) | 782 (18.5) | 508 (21.3) | 555 (28.4) |  |
| Age at Diagnosis, years | <55 | 20009 (21.1) | 2863 (25.8) | 1209 (28.6) | 634 (26.6) | 481 (24.6) | <0.001 |
|  | 55-59 | 11236 (11.8) | 1584 (14.3) | 553 (13.1) | 319 (13.4) | 264 (13.5) |  |
|  | 60-64 | 12141 (12.8) | 1521 (13.7) | 570 (13.5) | 356 (14.9) | 260 (13.3) |  |
|  | 65-69 | 12686 (13.4) | 1515 (13.6) | 586 (13.9) | 362 (15.2) | 265 (13.6) |  |
|  | 70-74 | 11236 (11.8) | 1211 (10.9) | 436 (10.3) | 270 (11.3) | 211 (10.8) |  |
|  | 75-79 | 10739 (11.3) | 1051 (9.5) | 413 (9.8) | 213 (8.9) | 199 (10.2) |  |
|  | 80-84 | 8848 (9.3) | 761 (6.8) | 264 (6.2) | 129 (5.4) | 149 (7.6) |  |
|  | 85+ | 8078 (8.5) | 606 (5.5) | 195 (4.6) | 103 (4.3) | 123 (6.3) |  |
| Sex | Male | 56041 (59.0) | 6225 (56.0) | 2565 (60.7) | 1395 (58.5) | 1158 (59.3) | <0.001 |
|  | Female | 38932 (41.0) | 4887 (44.0) | 1661 (39.3) | 991 (41.5) | 794 (40.7) |  |
| CDCC Score | 0 | 70718 (74.5) | 8113 (73.0) | 3171 (75.0) | 1850 (77.5) | 1545 (79.1) | <0.001 |
|  | 1 | 17484 (18.4) | 2159 (19.4) | 819 (19.4) | 428 (17.9) | 300 (15.4) |  |
|  | 2 | 4764 (5.0) | 527 (4.7) | 164 (3.9) | 74 (3.1) | 73 (3.7) |  |
|  | 3 | 2007 (2.1) | 313 (2.8) | 72 (1.7) | 34 (1.4) | 34 (1.7) |  |
| Insurance Status | Uninsured | 2270 (2.4) | 588 (5.3) | 329 (7.8) | 134 (5.6) | 74 (3.8) | <0.001 |
|  | Private Insurance | 38521 (40.6) | 3962 (35.7) | 1540 (36.4) | 1077 (45.1) | 862 (44.2) |  |
|  | Medicaid | 3683 (3.9) | 1243 (11.2) | 643 (15.2) | 318 (13.3) | 149 (7.6) |  |
|  | Medicare | 48190 (50.7) | 4879 (43.9) | 1618 (38.3) | 777 (32.6) | 755 (38.7) |  |
|  | Other/Unknown | 2309 (2.4) | 440 (4.0) | 96 (2.3) | 80 (3.4) | 112 (5.7) |  |
| Education Level | >21% | 11043 (11.6) | 3613 (32.5) | 1824 (43.2) | 428 (17.9) | 357 (18.3) | <0.001 |
|  | 13%-20.9% | 25255 (26.6) | 4235 (38.1) | 1126 (26.6) | 539 (22.6) | 522 (26.7) |  |
|  | 7%-12.9% | 34732 (36.6) | 2245 (20.2) | 872 (20.6) | 708 (29.7) | 586 (30.0) |  |
|  | <7% | 23453 (24.7) | 956 (8.6) | 391 (9.3) | 697 (29.2) | 466 (23.9) |  |
|  | Unknown | 490 (0.5) | 63 (0.6) | 13 (0.3) | 14 (0.6) | 21 (1.1) |  |
| Median Income | </=$47,999 | 35445 (37.3) | 7197 (64.8) | 2123 (50.2) | 598 (25.1) | 677 (34.7) | <0.001 |
|  | $48,000-$62,999 | 25412 (26.8) | 2030 (18.3) | 1126 (26.6) | 535 (22.4) | 508 (26.0) |  |
|  | $63,000 + | 33573 (35.4) | 1818 (16.4) | 963 (22.8) | 1239 (51.9) | 745 (38.2) |  |
|  | Unknown | 543 (0.6) | 67 (0.6) | 14 (0.3) | 14 (0.6) | 22 (1.1) |  |
| Residence | Metro | 78854 (83.0) | 10099 (90.9) | 4100 (97.0) | 2337 (97.9) | 1692 (86.7) | <0.001 |
|  | Urban/Rural | 16119 (17.0) | 1013 (9.1) | 126 (3.0) | 49 (2.1) | 260 (13.3) |  |
| AJCC Clinical T Stage | T0-1 | 16497 (17.4) | 1927 (17.3) | 804 (19.0) | 454 (19.0) | 373 (19.1) | <0.001 |
|  | T2 | 9355 (9.9) | 980 (8.8) | 354 (8.4) | 222 (9.3) | 170 (8.7) |  |
|  | T3-4 | 36301 (38.2) | 4406 (39.7) | 1734 (41.0) | 1025 (43.0) | 633 (32.4) |  |
|  | Tx | 32820 (34.6) | 3799 (34.2) | 1334 (31.6) | 685 (28.7) | 776 (39.8) |  |
| AJCC Clinical N Stage | N0 | 50913 (53.6) | 5749 (51.7) | 2137 (50.6) | 1281 (53.7) | 986 (50.5) | <0.001 |
|  | N+ | 15151 (16.0) | 1978 (17.8) | 863 (20.4) | 426 (17.9) | 266 (13.6) |  |
|  | Nx | 24457 (25.8) | 2844 (25.6) | 978 (23.1) | 487 (20.4) | 598 (30.6) |  |
|  | (Missing) | 4452 (4.7) | 541 (4.9) | 248 (5.9) | 192 (8.0) | 102 (5.2) |  |

Supplementary Table 7 Clinicopathologic characteristics of patients with non-metastatic breast cancers by race

|  |  | White | Black | Hispanic | Asian | Other | p-value |
| --- | --- | --- | --- | --- | --- | --- | --- |
| Centre Volume Quintile | Quintile 1 | 284517 (20.3) | 39448 (19.7) | 17979 (19.5) | 10213 (18.5) | 5868 (16.8) | <0.001 |
|  | Quintile 2 | 287442 (20.5) | 34843 (17.4) | 18965 (20.5) | 9660 (17.5) | 5263 (15.1) |  |
|  | Quintile 3 | 287024 (20.5) | 38159 (19.1) | 15390 (16.7) | 9371 (17.0) | 6912 (19.8) |  |
|  | Quintile 4 | 272798 (19.5) | 47001 (23.5) | 18601 (20.1) | 10824 (19.6) | 6937 (19.9) |  |
|  | Quintile 5 | 268561 (19.2) | 40517 (20.3) | 21467 (23.2) | 15128 (27.4) | 9858 (28.3) |  |
| Facility Type | Community | 789706 (56.4) | 82791 (41.4) | 40383 (43.7) | 25754 (46.7) | 15018 (43.1) | <0.001 |
|  | Academic | 355226 (25.4) | 73634 (36.8) | 31078 (33.6) | 19209 (34.8) | 12087 (34.7) |  |
|  | Others | 255410 (18.2) | 43543 (21.8) | 20941 (22.7) | 10233 (18.5) | 7733 (22.2) |  |
| Facility Location | Northeast | 286929 (20.5) | 31121 (15.6) | 16517 (17.9) | 10701 (19.4) | 7766 (22.3) | <0.001 |
|  | South | 470386 (33.6) | 106786 (53.4) | 32839 (35.5) | 9527 (17.3) | 9770 (28.0) |  |
|  | Midwest | 370634 (26.5) | 37511 (18.8) | 7239 (7.8) | 5656 (10.2) | 6470 (18.6) |  |
|  | West | 272393 (19.5) | 24550 (12.3) | 35807 (38.8) | 29312 (53.1) | 10832 (31.1) |  |
| Age at Diagnosis, years | <55 | 439157 (31.4) | 82684 (41.3) | 45189 (48.9) | 26386 (47.8) | 14663 (42.1) | <0.001 |
|  | 55-59 | 177177 (12.7) | 28234 (14.1) | 11932 (12.9) | 7321 (13.3) | 4720 (13.5) |  |
|  | 60-64 | 188462 (13.5) | 25856 (12.9) | 10522 (11.4) | 6634 (12.0) | 4552 (13.1) |  |
|  | 65-69 | 184771 (13.2) | 22257 (11.1) | 9225 (10.0) | 5720 (10.4) | 3943 (11.3) |  |
|  | 70-74 | 148171 (10.6) | 16312 (8.2) | 6378 (6.9) | 3976 (7.2) | 2777 (8.0) |  |
|  | 75-79 | 117401 (8.4) | 11875 (5.9) | 4673 (5.1) | 2601 (4.7) | 1884 (5.4) |  |
|  | 80-84 | 83351 (6.0) | 7364 (3.7) | 2755 (3.0) | 1537 (2.8) | 1328 (3.8) |  |
|  | 85+ | 61852 (4.4) | 5386 (2.7) | 1728 (1.9) | 1021 (1.8) | 971 (2.8) |  |
| Sex | Male | 14061 (1.0) | 2162 (1.1) | 632 (0.7) | 337 (0.6) | 355 (1.0) | <0.001 |
|  | Female | 1386281 (99.0) | 197806 (98.9) | 91770 (99.3) | 54859 (99.4) | 34483 (99.0) |  |
| CDCC Score | 0 | 1191978 (85.1) | 157793 (78.9) | 78107 (84.5) | 48865 (88.5) | 30212 (86.7) | <0.001 |
|  | 1 | 165048 (11.8) | 32299 (16.2) | 11755 (12.7) | 5392 (9.8) | 3676 (10.6) |  |
|  | 2 | 32789 (2.3) | 6977 (3.5) | 1868 (2.0) | 704 (1.3) | 730 (2.1) |  |
|  | 3 | 10527 (0.8) | 2899 (1.4) | 672 (0.7) | 235 (0.4) | 220 (0.6) |  |
| Insurance Status | Uninsured | 17352 (1.2) | 6950 (3.5) | 7718 (8.4) | 1771 (3.2) | 989 (2.8) | <0.001 |
|  | Private Insurance | 745665 (53.2) | 98064 (49.0) | 42981 (46.5) | 34699 (62.9) | 19110 (54.9) |  |
|  | Medicaid | 54379 (3.9) | 24341 (12.2) | 16378 (17.7) | 5839 (10.6) | 2795 (8.0) |  |
|  | Medicare | 546483 (39.0) | 63531 (31.8) | 21247 (23.0) | 11302 (20.5) | 9087 (26.1) |  |
|  | Other/Unknown | 36463 (2.6) | 7082 (3.5) | 4078 (4.4) | 1585 (2.9) | 2857 (8.2) |  |
| Education Level | >21% | 147413 (10.5) | 60487 (30.2) | 41497 (44.9) | 9240 (16.7) | 5326 (15.3) | <0.001 |
|  | 13%-20.9% | 315097 (22.5) | 71601 (35.8) | 21207 (23.0) | 10867 (19.7) | 7702 (22.1) |  |
|  | 7%-12.9% | 495283 (35.4) | 46557 (23.3) | 18371 (19.9) | 16932 (30.7) | 10950 (31.4) |  |
|  | <7% | 436307 (31.2) | 20401 (10.2) | 10897 (11.8) | 17930 (32.5) | 10556 (30.3) |  |
|  | Unknown | 6242 (0.4) | 922 (0.5) | 430 (0.5) | 227 (0.4) | 304 (0.9) |  |
| Median Income | </=$47,999 | 465504 (33.2) | 121358 (60.7) | 42680 (46.2) | 9418 (17.1) | 10545 (30.3) | <0.001 |
|  | $48,000-$62,999 | 385807 (27.6) | 41560 (20.8) | 25316 (27.4) | 12836 (23.3) | 8899 (25.5) |  |
|  | $63,000 + | 542181 (38.7) | 36057 (18.0) | 23951 (25.9) | 32709 (59.3) | 15072 (43.3) |  |
|  | Unknown | 6850 (0.5) | 993 (0.5) | 455 (0.5) | 233 (0.4) | 322 (0.9) |  |
| Residence | Metro | 1188495 (84.9) | 184792 (92.4) | 88570 (95.9) | 54049 (97.9) | 30787 (88.4) | <0.001 |
|  | Urban/Rural | 211847 (15.1) | 15176 (7.6) | 3832 (4.1) | 1147 (2.1) | 4051 (11.6) |  |
| AJCC Clinical T Stage | T0-1 | 758759 (54.2) | 91440 (45.7) | 42526 (46.0) | 27717 (50.2) | 16499 (47.4) | <0.001 |
|  | T2 | 236474 (16.9) | 44817 (22.4) | 21039 (22.8) | 11855 (21.5) | 6614 (19.0) |  |
|  | T3-4 | 60717 (4.3) | 17094 (8.5) | 6777 (7.3) | 3127 (5.7) | 1931 (5.5) |  |
|  | Tx | 344392 (24.6) | 46617 (23.3) | 22060 (23.9) | 12497 (22.6) | 9794 (28.1) |  |
| AJCC Clinical N Stage | N0 | 938438 (67.0) | 120157 (60.1) | 56455 (61.1) | 37157 (67.3) | 21221 (60.9) | <0.001 |
|  | N+ | 143442 (10.2) | 36599 (18.3) | 15730 (17.0) | 7198 (13.0) | 4470 (12.8) |  |
|  | Nx | 318462 (22.7) | 43212 (21.6) | 20217 (21.9) | 10841 (19.6) | 9147 (26.3) |  |

Supplementary Table 8 Clinicopathologic characteristics of patients with non-metastatic lung cancers by race

|  |  | White | Black | Hispanic | Asian | Other | p-value |
| --- | --- | --- | --- | --- | --- | --- | --- |
| Centre Volume Quintile | Quintile 1 | 164666 (20.2) | 19096 (20.0) | 6044 (26.0) | 4215 (26.1) | 2417 (18.2) | <0.001 |
|  | Quintile 2 | 166054 (20.4) | 19150 (20.1) | 4603 (19.8) | 3292 (20.4) | 1940 (14.6) |  |
|  | Quintile 3 | 162176 (19.9) | 17998 (18.9) | 4983 (21.4) | 3216 (19.9) | 3038 (22.8) |  |
|  | Quintile 4 | 161282 (19.8) | 20371 (21.4) | 4480 (19.2) | 3028 (18.7) | 2875 (21.6) |  |
|  | Quintile 5 | 161174 (19.8) | 18689 (19.6) | 3173 (13.6) | 2410 (14.9) | 3045 (22.9) |  |
| Facility Type | Community | 461133 (56.6) | 39944 (41.9) | 10881 (46.7) | 7039 (43.6) | 5759 (43.3) | <0.001 |
|  | Academic | 236993 (29.1) | 40778 (42.8) | 8585 (36.9) | 7167 (44.3) | 5548 (41.7) |  |
|  | Others | 117226 (14.4) | 14582 (15.3) | 3817 (16.4) | 1955 (12.1) | 2008 (15.1) |  |
| Facility Location | Northeast | 166987 (20.5) | 14804 (15.5) | 4839 (20.8) | 3678 (22.8) | 3014 (22.6) | <0.001 |
|  | South | 320248 (39.3) | 53293 (55.9) | 9951 (42.7) | 2769 (17.1) | 3671 (27.6) |  |
|  | Midwest | 226175 (27.7) | 22074 (23.2) | 1899 (8.2) | 1609 (10.0) | 3362 (25.2) |  |
|  | West | 101942 (12.5) | 5133 (5.4) | 6594 (28.3) | 8105 (50.2) | 3268 (24.5) |  |
| Age at Diagnosis, years | <55 | 71279 (8.7) | 13915 (14.6) | 2627 (11.3) | 1776 (11.0) | 1542 (11.6) | <0.001 |
|  | 55-59 | 71165 (8.7) | 12699 (13.3) | 2107 (9.0) | 1423 (8.8) | 1383 (10.4) |  |
|  | 60-64 | 103776 (12.7) | 15046 (15.8) | 3006 (12.9) | 1978 (12.2) | 1865 (14.0) |  |
|  | 65-69 | 142476 (17.5) | 16573 (17.4) | 4011 (17.2) | 2676 (16.6) | 2348 (17.6) |  |
|  | 70-74 | 151304 (18.6) | 15003 (15.7) | 4130 (17.7) | 2721 (16.8) | 2343 (17.6) |  |
|  | 75-79 | 135722 (16.6) | 11768 (12.3) | 3795 (16.3) | 2679 (16.6) | 1931 (14.5) |  |
|  | 80-84 | 92352 (11.3) | 6891 (7.2) | 2375 (10.2) | 1839 (11.4) | 1272 (9.6) |  |
|  | 85+ | 47278 (5.8) | 3409 (3.6) | 1232 (5.3) | 1069 (6.6) | 631 (4.7) |  |
| Sex | Male | 419344 (51.4) | 50351 (52.8) | 13227 (56.8) | 9155 (56.6) | 7131 (53.6) | <0.001 |
|  | Female | 396008 (48.6) | 44953 (47.2) | 10056 (43.2) | 7006 (43.4) | 6184 (46.4) |  |
| CDCC Score | 0 | 451700 (55.4) | 53267 (55.9) | 13636 (58.6) | 11328 (70.1) | 8338 (62.6) | <0.001 |
|  | 1 | 239339 (29.4) | 26194 (27.5) | 6234 (26.8) | 3464 (21.4) | 3363 (25.3) |  |
|  | 2 | 88929 (10.9) | 10525 (11.0) | 2360 (10.1) | 969 (6.0) | 1151 (8.6) |  |
|  | 3 | 35384 (4.3) | 5318 (5.6) | 1053 (4.5) | 400 (2.5) | 463 (3.5) |  |
| Insurance Status | Uninsured | 16390 (2.0) | 4393 (4.6) | 1217 (5.2) | 538 (3.3) | 375 (2.8) | <0.001 |
|  | Private Insurance | 205000 (25.1) | 22999 (24.1) | 5562 (23.9) | 4911 (30.4) | 3570 (26.8) |  |
|  | Medicaid | 35291 (4.3) | 11362 (11.9) | 2703 (11.6) | 1843 (11.4) | 975 (7.3) |  |
|  | Medicare | 528968 (64.9) | 52417 (55.0) | 12855 (55.2) | 8417 (52.1) | 7090 (53.2) |  |
|  | Other/Unknown | 29703 (3.6) | 4133 (4.3) | 946 (4.1) | 452 (2.8) | 1305 (9.8) |  |
| Education Level | >21% | 122269 (15.0) | 35897 (37.7) | 11009 (47.3) | 4073 (25.2) | 2414 (18.1) | <0.001 |
|  | 13%-20.9% | 228677 (28.0) | 35010 (36.7) | 5552 (23.8) | 3535 (21.9) | 3626 (27.2) |  |
|  | 7%-12.9% | 284619 (34.9) | 17789 (18.7) | 4409 (18.9) | 4770 (29.5) | 4348 (32.7) |  |
|  | <7% | 175864 (21.6) | 6264 (6.6) | 2200 (9.4) | 3723 (23.0) | 2842 (21.3) |  |
|  | Unknown | 3923 (0.5) | 344 (0.4) | 113 (0.5) | 60 (0.4) | 85 (0.6) |  |
| Median Income | </=$47,999 | 354213 (43.4) | 67645 (71.0) | 12159 (52.2) | 3931 (24.3) | 5585 (41.9) | <0.001 |
|  | $48,000-$62,999 | 225419 (27.6) | 16200 (17.0) | 6110 (26.2) | 4289 (26.5) | 3570 (26.8) |  |
|  | $63,000 + | 231373 (28.4) | 11069 (11.6) | 4894 (21.0) | 7877 (48.7) | 4068 (30.6) |  |
|  | Unknown | 4347 (0.5) | 390 (0.4) | 120 (0.5) | 64 (0.4) | 92 (0.7) |  |
| Residence | Metro | 652766 (80.1) | 85539 (89.8) | 21856 (93.9) | 15723 (97.3) | 10559 (79.3) | <0.001 |
|  | Urban/Rural | 162586 (19.9) | 9765 (10.2) | 1427 (6.1) | 438 (2.7) | 2756 (20.7) |  |
| AJCC Clinical T Stage | T0-1 | 237128 (29.1) | 23879 (25.1) | 5816 (25.0) | 4495 (27.8) | 3404 (25.6) | <0.001 |
|  | T2 | 105752 (13.0) | 12486 (13.1) | 2753 (11.8) | 1866 (11.5) | 1599 (12.0) |  |
|  | T3-4 | 269904 (33.1) | 36902 (38.7) | 8402 (36.1) | 5509 (34.1) | 4278 (32.1) |  |
|  | Tx | 202568 (24.8) | 22037 (23.1) | 6312 (27.1) | 4291 (26.6) | 4034 (30.3) |  |
| AJCC Clinical N Stage | N0 | 347091 (42.6) | 37026 (38.9) | 9231 (39.6) | 6780 (42.0) | 5099 (38.3) | <0.001 |
|  | N+ | 281906 (34.6) | 37750 (39.6) | 8195 (35.2) | 5489 (34.0) | 4481 (33.7) |  |
|  | Nx | 186355 (22.9) | 20528 (21.5) | 5857 (25.2) | 3892 (24.1) | 3735 (28.1) |  |

Supplementary Table 9 Reasons for no surgery in patients non-metastatic cancers, stratified race

|  | **White** | **Black** | **Hispanic** | **Asian** | **Other** | **Total** | **p-value** |
| --- | --- | --- | --- | --- | --- | --- | --- |
| **Oesophagus** | | | | | | | |
| Physician Reccomendation | 40796 (84.9) | 6834 (87.1) | 2020 (88.9) | 1045 (87.2) | 934 (88.2) | 51629 (85.4) | <0.001 |
| Contraindicated | 5035 (10.5) | 718 (9.1) | 187 (8.2) | 105 (8.8) | 83 (7.8) | 6128 (10.1) |  |
| Patient Death | 442 (0.9) | 54 (0.7) | 8 (0.4) | 7 (0.6) | 3 (0.3) | 514 (0.9) |  |
| Patient Refused | 1782 (3.7) | 244 (3.1) | 56 (2.5) | 42 (3.5) | 39 (3.7) | 2163 (3.6) |  |
| **Stomach** | | | | | | |  |
| Physician Reccomendation | 21121 (81.4) | 3988 (77.9) | 2471 (85.1) | 1404 (81.7) | 713 (85.6) | 29697 (81.3) | <0.001 |
| Contraindicated | 3162 (12.2) | 633 (12.4) | 268 (9.2) | 168 (9.8) | 71 (8.5) | 4302 (11.8) |  |
| Patient Death | 296 (1.1) | 66 (1.3) | 23 (0.8) | 10 (0.6) | 13 (1.6) | 408 (1.1) |  |
| Patient Refused | 1384 (5.3) | 434 (8.5) | 143 (4.9) | 137 (8.0) | 36 (4.3) | 2134 (5.8) |  |
| **Liver** |  |  |  |  |  |  |  |
| Physician Reccomendation | 57241 (88.6) | 14760 (87.9) | 12381 (90.4) | 5917 (89.3) | 2833 (89.5) | 93132 (88.8) | <0.001 |
| Contraindicated | 6135 (9.5) | 1654 (9.9) | 1131 (8.3) | 554 (8.4) | 285 (9.0) | 9759 (9.3) |  |
| Patient Death | 370 (0.6) | 142 (0.8) | 59 (0.4) | 33 (0.5) | 16 (0.5) | 620 (0.6) |  |
| Patient Refused | 829 (1.3) | 234 (1.4) | 124 (0.9) | 119 (1.8) | 33 (1.0) | 1339 (1.3) |  |
| **Pancreas** |  |  |  |  |  |  |  |
| Physician Reccomendation | 72693 (85.0) | 11544 (84.5) | 4811 (88.8) | 2340 (87.8) | 2295 (89.2) | 93683 (85.3) | <0.001 |
| Contraindicated | 10071 (11.8) | 1754 (12.8) | 485 (9.0) | 253 (9.5) | 219 (8.5) | 12782 (11.6) |  |
| Patient Death | 513 (0.6) | 78 (0.6) | 21 (0.4) | 10 (0.4) | 12 (0.5) | 634 (0.6) |  |
| Patient Refused | 2247 (2.6) | 293 (2.1) | 100 (1.8) | 61 (2.3) | 47 (1.8) | 2748 (2.5) |  |
| **Colon** |  |  |  |  |  |  |  |
| Physician Reccomendation | 25687 (73.9) | 4861 (73.6) | 2040 (83.2) | 984 (78.6) | 968 (85.6) | 34540 (74.8) | <0.001 |
| Contraindicated | 4064 (11.7) | 687 (10.4) | 191 (7.8) | 111 (8.9) | 72 (6.4) | 5125 (11.1) |  |
| Patient Death | 842 (2.4) | 115 (1.7) | 30 (1.2) | 12 (1.0) | 16 (1.4) | 1015 (2.2) |  |
| Patient Refused | 4158 (12.0) | 938 (14.2) | 191 (7.8) | 145 (11.6) | 75 (6.6) | 5507 (11.9) |  |
| **Rectal** |  |  |  |  |  |  |  |
| Physician Reccomendation | 11530 (76.0) | 1881 (76.2) | 602 (84.2) | 303 (79.7) | 386 (86.7) | 14702 (76.6) | <0.001 |
| Contraindicated | 1560 (10.3) | 219 (8.9) | 40 (5.6) | 23 (6.1) | 23 (5.2) | 1865 (9.7) |  |
| Patient Death | 340 (2.2) | 33 (1.3) | 5 (0.7) | 3 (0.8) | 2 (0.4) | 383 (2.0) |  |
| Patient Refused | 1748 (11.5) | 336 (13.6) | 68 (9.5) | 51 (13.4) | 34 (7.6) | 2237 (11.7) |  |
| **Lung** |  |  |  |  |  |  |  |
| Physician Reccomendation | 452730 (87.8) | 59150 (87.8) | 13806 (90.8) | 8636 (90.3) | 7981 (89.0) | 542303 (87.9) | <0.001 |
| Contraindicated | 50861 (9.9) | 6276 (9.3) | 1081 (7.1) | 639 (6.7) | 759 (8.5) | 59616 (9.7) |  |
| Patient Death | 1724 (0.3) | 244 (0.4) | 59 (0.4) | 30 (0.3) | 38 (0.4) | 2095 (0.3) |  |
| Patient Refused | 10388 (2.0) | 1703 (2.5) | 251 (1.7) | 258 (2.7) | 188 (2.1) | 12788 (2.1) |  |
| **Breast** |  |  |  |  |  |  |  |
| Physician Reccomendation | 48539 (81.2) | 11884 (81.1) | 5639 (90.9) | 2480 (85.5) | 3092 (90.7) | 71634 (82.4) | <0.001 |
| Contraindicated | 4071 (6.8) | 809 (5.5) | 172 (2.8) | 82 (2.8) | 74 (2.2) | 5208 (6.0) |  |
| Patient Death | 775 (1.3) | 201 (1.4) | 40 (0.6) | 25 (0.9) | 19 (0.6) | 1060 (1.2) |  |
| Patient Refused | 6401 (10.7) | 1765 (12.0) | 351 (5.7) | 312 (10.8) | 223 (6.5) | 9052 (10.4) |  |

Supplementary Table 10 Impact of race on receipt of curative cancer surgery in non-metastatic cancers, stratified by time cohorts

|  | **2004 - 2007** | | **2008 - 2011** | | **2012 - 2016** | |
| --- | --- | --- | --- | --- | --- | --- |
|  | **Patients, n** | **OR (95% CI)** | **Patients, n** | **OR (95% CI)** | **Patients, n** | **OR (95% CI)** |
| **Oesophagus** |  |  |  |  |  |  |
| White | 10490 (43.3) | REF | 11333 (43.9) | REF | 15573 (43.9) | REF |
| Black | 600 (20.3) | 0.33 (0.30-0.37, p<0.001) | 521 (17.9) | 0.28 (0.25-0.31, p<0.001) | 675 (17.8) | 0.28 (0.25-0.30, p<0.001) |
| Hispanic | 296 (33.9) | 0.67 (0.58-0.77, p<0.001) | 345 (34.1) | 0.66 (0.58-0.75, p<0.001) | 479 (31.8) | 0.60 (0.53-0.66, p<0.001) |
| Asian | 120 (29.5) | 0.55 (0.44-0.68, p<0.001) | 145 (29.6) | 0.54 (0.44-0.65, p<0.001) | 267 (32.0) | 0.60 (0.52-0.70, p<0.001) |
| Other | 272 (45.5) | 1.09 (0.93-1.28, p=0.292) | 222 (40.7) | 0.87 (0.73-1.04, p=0.126) | 261 (39.0) | 0.81 (0.70-0.95, p=0.010) |
| **Stomach** |  |  |  |  |  |  |
| White | 12065 (61.1) |  | 11975 (60.9) |  | 15591 (59.5) |  |
| Black | 2470 (62.8) | 1.07 (1.00-1.15, p=0.056) | 2560 (62.6) | 1.08 (1.00-1.15, p=0.040) | 3166 (59.8) | 1.01 (0.95-1.08, p=0.692) |
| Hispanic | 1425 (65.5) | 1.21 (1.10-1.32, p<0.001) | 1627 (63.9) | 1.14 (1.05-1.24, p=0.003) | 2303 (65.1) | 1.27 (1.18-1.36, p<0.001) |
| Asian | 1280 (73.4) | 1.75 (1.57-1.96, p<0.001) | 1329 (75.0) | 1.93 (1.73-2.16, p<0.001) | 1919 (70.3) | 1.61 (1.47-1.75, p<0.001) |
| Other | 370 (58.9) | 0.91 (0.78-1.07, p=0.263) | 366 (59.0) | 0.93 (0.79-1.09, p=0.356) | 546 (63.0) | 1.16 (1.01-1.33, p=0.043) |
| **Liver** |  |  |  |  |  |  |
| White | 6904 (35.4) | - | 8574 (31.2) | - | 13469 (28.9) | - |
| Black | 1124 (26.1) | 0.65 (0.60-0.70, p<0.001) | 1744 (25.6) | 0.76 (0.71-0.80, p<0.001) | 2859 (25.1) | 0.82 (0.78-0.86, p<0.001) |
| Hispanic | 1117 (31.4) | 0.84 (0.77-0.90, p<0.001) | 1366 (25.1) | 0.74 (0.69-0.79, p<0.001) | 2195 (23.4) | 0.75 (0.71-0.79, p<0.001) |
| Asian | 1015 (39.8) | 1.21 (1.11-1.32, p<0.001) | 1260 (38.2) | 1.36 (1.26-1.46, p<0.001) | 1908 (38.5) | 1.54 (1.45-1.63, p<0.001) |
| Other | 323 (34.1) | 0.94 (0.82-1.08, p=0.416) | 408 (29.7) | 0.93 (0.83-1.05, p=0.231) | 599 (27.5) | 0.93 (0.85-1.03, p=0.160) |
| **Pancreas** |  |  |  |  |  |  |
| White | 12307 (33.5) | - | 15637 (38.4) | - | 24026 (40.0) | - |
| Black | 1347 (27.1) | 0.74 (0.69-0.79, p<0.001) | 1797 (31.2) | 0.73 (0.69-0.77, p<0.001) | 2869 (32.0) | 0.71 (0.67-0.74, p<0.001) |
| Hispanic | 588 (31.5) | 0.91 (0.83-1.01, p=0.073) | 796 (32.9) | 0.79 (0.72-0.86, p<0.001) | 1478 (37.0) | 0.88 (0.82-0.94, p<0.001) |
| Asian | 264 (28.9) | 0.81 (0.70-0.93, p=0.004) | 436 (36.5) | 0.92 (0.82-1.04, p=0.191) | 754 (37.5) | 0.90 (0.82-0.98, p=0.022) |
| Other | 305 (27.2) | 0.74 (0.65-0.84, p<0.001) | 336 (29.9) | 0.68 (0.60-0.78, p<0.001) | 508 (34.5) | 0.79 (0.71-0.88, p<0.001) |
| **Colon** |  |  |  |  |  |  |
| White | 204541 (94.5) | - | 181088 (94.6) | - | 216762 (94.5) | - |
| Black | 27383 (92.8) | 0.75 (0.71-0.78, p<0.001) | 26327 (93.0) | 0.76 (0.72-0.80, p<0.001) | 33365 (93.0) | 0.77 (0.74-0.81, p<0.001) |
| Hispanic | 10033 (93.7) | 0.86 (0.80-0.94, p<0.001) | 11092 (94.0) | 0.90 (0.83-0.97, p=0.008) | 15896 (93.6) | 0.86 (0.81-0.92, p<0.001) |
| Asian | 5347 (94.3) | 0.95 (0.85-1.07, p=0.402) | 5866 (94.3) | 0.94 (0.85-1.05, p=0.288) | 9142 (94.1) | 0.93 (0.85-1.01, p=0.092) |
| Other | 3936 (91.4) | 0.62 (0.55-0.69, p<0.001) | 3587 (91.5) | 0.61 (0.55-0.69, p<0.001) | 4651 (91.6) | 0.63 (0.57-0.70, p<0.001) |
| **Rectal** |  |  |  |  |  |  |
| White | 26694 (86.1) | - | 23443 (84.4) | - | 29659 (81.9) | - |
| Black | 2558 (79.1) | 0.61 (0.56-0.67, p<0.001) | 2712 (77.9) | 0.65 (0.60-0.71, p<0.001) | 3373 (76.7) | 0.73 (0.67-0.78, p<0.001) |
| Hispanic | 1010 (85.5) | 0.95 (0.81-1.13, p=0.564) | 1074 (84.0) | 0.97 (0.83-1.13, p=0.676) | 1427 (80.8) | 0.93 (0.82-1.05, p=0.230) |
| Asian | 482 (84.3) | 0.86 (0.69-1.09, p=0.207) | 552 (83.3) | 0.92 (0.75-1.13, p=0.421) | 972 (84.4) | 1.20 (1.02-1.41, p=0.029) |
| Other | 497 (78.0) | 0.57 (0.47-0.69, p<0.001) | 468 (78.9) | 0.69 (0.57-0.85, p<0.001) | 542 (75.1) | 0.66 (0.56-0.79, p<0.001) |
| **Breast** |  |  |  |  |  |  |
| White | 372367 (96.4) | - | 412475 (95.9) | - | 555714 (95.2) | - |
| Black | 46227 (93.8) | 0.57 (0.55-0.59, p<0.001) | 56768 (93.0) | 0.56 (0.54-0.58, p<0.001) | 82314 (91.8) | 0.57 (0.56-0.59, p<0.001) |
| Hispanic | 19382 (93.8) | 0.57 (0.54-0.61, p<0.001) | 25566 (93.3) | 0.59 (0.56-0.62, p<0.001) | 41252 (93.0) | 0.68 (0.65-0.71, p<0.001) |
| Asian | 11292 (95.9) | 0.88 (0.81-0.97, p=0.009) | 15028 (94.5) | 0.73 (0.68-0.78, p<0.001) | 25977 (94.4) | 0.86 (0.81-0.90, p<0.001) |
| Other | 8538 (91.5) | 0.41 (0.38-0.44, p<0.001) | 9750 (89.3) | 0.35 (0.33-0.38, p<0.001) | 13142 (90.1) | 0.46 (0.44-0.49, p<0.001) |
| **Lung** |  |  |  |  |  |  |
| White | 89464 (34.7) | - | 92239 (37.6) | - | 117946 (37.7) | - |
| Black | 7495 (26.5) | 0.68 (0.66-0.70, p<0.001) | 8747 (30.4) | 0.72 (0.70-0.74, p<0.001) | 11689 (30.6) | 0.73 (0.71-0.75, p<0.001) |
| Hispanic | 1848 (29.5) | 0.79 (0.74-0.83, p<0.001) | 2453 (34.2) | 0.86 (0.82-0.91, p<0.001) | 3785 (38.4) | 1.03 (0.99-1.08, p=0.136) |
| Asian | 1483 (35.0) | 1.01 (0.95-1.08, p=0.736) | 1825 (39.3) | 1.07 (1.01-1.14, p=0.023) | 3290 (45.2) | 1.36 (1.30-1.43, p<0.001) |
| Other | 1291 (30.0) | 0.81 (0.75-0.86, p<0.001) | 1335 (32.1) | 0.78 (0.73-0.84, p<0.001) | 1723 (35.5) | 0.91 (0.86-0.97, p=0.002) |

Supplementary Table 11 Impact of race on receipt of neoadjuvant therapy in non-metastatic cancers, stratified by time cohorts

|  | **2004 - 2007** | | **2008 - 2011** | | **2012 - 2016** | |
| --- | --- | --- | --- | --- | --- | --- |
|  | **Patients, n** | **OR (95% CI)** | **Patients, n** | **OR (95% CI)** | **Patients, n** | **OR (95% CI)** |
| **Oesophagus** |  |  |  |  |  |  |
| White | 4436 (18.3) | REF | 5850 (22.7) | REF | 9747 (27.5) | REF |
| Black | 267 (9.1) | 0.44 (0.39-0.50, p<0.001) | 272 (9.3) | 0.35 (0.31-0.40, p<0.001) | 438 (11.6) | 0.34 (0.31-0.38, p<0.001) |
| Hispanic | 118 (13.5) | 0.70 (0.57-0.84, p<0.001) | 179 (17.7) | 0.73 (0.62-0.86, p<0.001) | 315 (20.9) | 0.70 (0.61-0.79, p<0.001) |
| Asian | 53 (13.0) | 0.67 (0.49-0.88, p=0.006) | 82 (16.7) | 0.69 (0.54-0.86, p=0.002) | 181 (21.7) | 0.73 (0.62-0.86, p<0.001) |
| Other | 104 (17.4) | 0.94 (0.75-1.16, p=0.562) | 90 (16.5) | 0.67 (0.53-0.84, p=0.001) | 137 (20.4) | 0.68 (0.56-0.82, p<0.001) |
| **Stomach** |  |  |  |  |  |  |
| White | 1924 (9.7) | REF | 3608 (18.3) | REF | 6956 (26.6) | REF |
| Black | 155 (3.9) | 0.38 (0.32-0.45, p<0.001) | 385 (9.4) | 0.46 (0.41-0.52, p<0.001) | 850 (16.1) | 0.53 (0.49-0.57, p<0.001) |
| Hispanic | 107 (4.9) | 0.48 (0.39-0.58, p<0.001) | 333 (13.1) | 0.67 (0.59-0.76, p<0.001) | 732 (20.7) | 0.72 (0.66-0.79, p<0.001) |
| Asian | 73 (4.2) | 0.40 (0.32-0.51, p<0.001) | 173 (9.8) | 0.48 (0.41-0.56, p<0.001) | 429 (15.7) | 0.52 (0.46-0.57, p<0.001) |
| Other | 43 (6.8) | 0.68 (0.49-0.92, p=0.016) | 78 (12.6) | 0.64 (0.50-0.81, p<0.001) | 197 (22.7) | 0.81 (0.69-0.95, p=0.012) |
| **Liver** |  |  |  |  |  |  |
| White | 888 (4.5) | REF | 2300 (8.4) | REF | 3724 (8.0) | REF |
| Black | 174 (4.0) | 0.88 (0.75-1.04, p=0.147) | 461 (6.8) | 0.79 (0.71-0.88, p<0.001) | 741 (6.5) | 0.80 (0.74-0.87, p<0.001) |
| Hispanic | 207 (5.8) | 1.30 (1.11-1.51, p=0.001) | 490 (9.0) | 1.08 (0.97-1.20, p=0.137) | 712 (7.6) | 0.95 (0.87-1.03, p=0.196) |
| Asian | 159 (6.2) | 1.40 (1.17-1.66, p<0.001) | 336 (10.2) | 1.24 (1.10-1.40, p<0.001) | 416 (8.4) | 1.05 (0.95-1.17, p=0.332) |
| Other | 48 (5.1) | 1.12 (0.82-1.49, p=0.459) | 125 (9.1) | 1.09 (0.90-1.32, p=0.348) | 185 (8.5) | 1.07 (0.91-1.24, p=0.397) |
| **Pancreas** |  |  |  |  |  |  |
| White | 1055 (2.9) | REF | 2248 (5.5) | REF | 6288 (10.5) | REF |
| Black | 115 (2.3) | 0.80 (0.66-0.97, p=0.026) | 270 (4.7) | 0.84 (0.74-0.96, p=0.009) | 653 (7.3) | 0.67 (0.62-0.73, p<0.001) |
| Hispanic | 35 (1.9) | 0.65 (0.45-0.89, p=0.012) | 90 (3.7) | 0.66 (0.53-0.82, p<0.001) | 297 (7.4) | 0.69 (0.61-0.77, p<0.001) |
| Asian | 22 (2.4) | 0.84 (0.53-1.25, p=0.408) | 50 (4.2) | 0.75 (0.56-0.99, p=0.047) | 159 (7.9) | 0.73 (0.62-0.86, p<0.001) |
| Other | 33 (2.9) | 1.02 (0.71-1.43, p=0.895) | 51 (4.5) | 0.81 (0.60-1.07, p=0.153) | 153 (10.4) | 0.99 (0.83-1.17, p=0.905) |
| **Colon** |  |  |  |  |  |  |
| White | 5643 (2.6) | REF | 4854 (2.5) | REF | 6106 (2.7) | REF |
| Black | 810 (2.7) | 1.05 (0.98-1.14, p=0.167) | 724 (2.6) | 1.01 (0.93-1.09, p=0.822) | 846 (2.4) | 0.88 (0.82-0.95, p=0.001) |
| Hispanic | 356 (3.3) | 1.28 (1.15-1.43, p<0.001) | 380 (3.2) | 1.28 (1.15-1.42, p<0.001) | 581 (3.4) | 1.30 (1.19-1.41, p<0.001) |
| Asian | 203 (3.6) | 1.39 (1.20-1.59, p<0.001) | 251 (4.0) | 1.62 (1.42-1.84, p<0.001) | 331 (3.4) | 1.29 (1.15-1.44, p<0.001) |
| Other | 165 (3.8) | 1.49 (1.27-1.74, p<0.001) | 138 (3.5) | 1.40 (1.17-1.66, p<0.001) | 191 (3.8) | 1.43 (1.23-1.65, p<0.001) |
| **Rectal** |  |  |  |  |  |  |
| White | 9615 (31.0) | REF | 10575 (38.1) | REF | 15097 (41.7) | REF |
| Black | 971 (30.0) | 0.95 (0.88-1.03, p=0.250) | 1249 (35.9) | 0.91 (0.85-0.98, p=0.012) | 1650 (37.5) | 0.84 (0.79-0.89, p<0.001) |
| Hispanic | 340 (28.8) | 0.90 (0.79-1.02, p=0.104) | 471 (36.8) | 0.95 (0.84-1.06, p=0.368) | 752 (42.6) | 1.04 (0.94-1.14, p=0.465) |
| Asian | 208 (36.4) | 1.27 (1.07-1.51, p=0.006) | 270 (40.7) | 1.12 (0.95-1.31, p=0.165) | 493 (42.8) | 1.05 (0.93-1.18, p=0.445) |
| Other | 138 (21.7) | 0.62 (0.51-0.74, p<0.001) | 207 (34.9) | 0.87 (0.73-1.03, p=0.116) | 256 (35.5) | 0.77 (0.66-0.89, p=0.001) |
| **Breast** |  |  |  |  |  |  |
| White | 18096 (4.7) | REF | 34984 (8.1) | REF | 62090 (10.6) | REF |
| Black | 3725 (7.6) | 1.66 (1.60-1.73, p<0.001) | 8613 (14.1) | 1.85 (1.81-1.90, p<0.001) | 15435 (17.2) | 1.75 (1.72-1.78, p<0.001) |
| Hispanic | 1650 (8.0) | 1.77 (1.68-1.86, p<0.001) | 3887 (14.2) | 1.87 (1.80-1.93, p<0.001) | 7182 (16.2) | 1.62 (1.58-1.67, p<0.001) |
| Asian | 649 (5.5) | 1.19 (1.09-1.29, p<0.001) | 1743 (11.0) | 1.39 (1.32-1.46, p<0.001) | 3546 (12.9) | 1.24 (1.20-1.29, p<0.001) |
| Other | 568 (6.1) | 1.32 (1.21-1.44, p<0.001) | 1180 (10.8) | 1.37 (1.29-1.45, p<0.001) | 2011 (13.8) | 1.34 (1.28-1.41, p<0.001) |
| **Lung** |  |  |  |  |  |  |
| White | 8475 (3.3) | REF | 7763 (3.2) | REF | 9426 (3.0) | REF |
| Black | 844 (3.0) | 0.90 (0.84-0.97, p=0.006) | 782 (2.7) | 0.85 (0.79-0.92, p<0.001) | 1097 (2.9) | 0.95 (0.89-1.01, p=0.125) |
| Hispanic | 210 (3.3) | 1.02 (0.88-1.17, p=0.799) | 214 (3.0) | 0.94 (0.82-1.08, p=0.384) | 365 (3.7) | 1.24 (1.11-1.38, p<0.001) |
| Asian | 166 (3.9) | 1.20 (1.02-1.40, p=0.024) | 179 (3.9) | 1.22 (1.05-1.42, p=0.009) | 301 (4.1) | 1.39 (1.23-1.56, p<0.001) |
| Other | 176 (4.1) | 1.25 (1.07-1.45, p=0.004) | 185 (4.4) | 1.42 (1.22-1.65, p<0.001) | 204 (4.2) | 1.41 (1.22-1.62, p<0.001) |

Supplementary Table 12 Impact of race on receipt of neoadjuvant therapy in non-metastatic cancers, stratified by time cohorts

|  | **2004 - 2007** | **2008 - 2011** | **2012 - 2016** |
| --- | --- | --- | --- |
|  | **HR (95% CI)** | **HR (95% CI)** | **HR (95% CI)** |
| **Oesophagus** |  |  |  |
| White | REF | REF | REF |
| Black | 1.12 (1.07-1.17, p<0.001) | 1.15 (1.09-1.20, p<0.001) | 1.15 (1.09-1.20, p<0.001) |
| Hispanic | 0.87 (0.80-0.94, p<0.001) | 0.85 (0.79-0.92, p<0.001) | 0.85 (0.79-0.92, p<0.001) |
| Asian | 0.75 (0.67-0.84, p<0.001) | 0.81 (0.72-0.90, p<0.001) | 0.81 (0.72-0.90, p<0.001) |
| Other | 0.98 (0.89-1.08, p=0.680) | 0.92 (0.83-1.02, p=0.131) | 0.92 (0.83-1.02, p=0.131) |
| **Stomach** |  |  |  |
| White | REF | REF | REF |
| Black | 0.99 (0.95-1.03, p=0.700) | 0.97 (0.93-1.01, p=0.117) | 0.97 (0.92-1.01, p=0.142) |
| Hispanic | 0.83 (0.78-0.88, p<0.001) | 0.78 (0.74-0.82, p<0.001) | 0.79 (0.75-0.85, p<0.001) |
| Asian | 0.69 (0.64-0.73, p<0.001) | 0.65 (0.61-0.69, p<0.001) | 0.61 (0.56-0.65, p<0.001) |
| Other | 0.89 (0.81-0.97, p=0.012) | 0.91 (0.82-1.00, p=0.061) | 0.78 (0.69-0.87, p<0.001) |
| **Liver** |  |  |  |
| White |  |  |  |
| Black | 1.14 (1.10-1.19, p<0.001) | 1.06 (1.02-1.09, p<0.001) | 0.99 (0.96-1.03, p=0.726) |
| Hispanic | 0.94 (0.90-0.98, p=0.002) | 0.90 (0.87-0.93, p<0.001) | 0.84 (0.81-0.87, p<0.001) |
| Asian | 0.81 (0.77-0.85, p<0.001) | 0.77 (0.74-0.81, p<0.001) | 0.75 (0.72-0.79, p<0.001) |
| Other | 0.96 (0.90-1.04, p=0.330) | 1.02 (0.95-1.08, p=0.609) | 0.84 (0.79-0.90, p<0.001) |
| **Pancreas** |  |  |  |
| White | - | - | - |
| Black | 1.06 (1.03-1.09, p<0.001) | 1.05 (1.02-1.08, p=0.002) | 1.02 (0.99-1.06, p=0.112) |
| Hispanic | 0.90 (0.86-0.95, p<0.001) | 0.89 (0.85-0.93, p<0.001) | 0.85 (0.82-0.89, p<0.001) |
| Asian | 0.98 (0.91-1.05, p=0.600) | 0.87 (0.82-0.93, p<0.001) | 0.87 (0.82-0.93, p<0.001) |
| Other | 1.04 (0.97-1.10, p=0.265) | 1.07 (1.00-1.14, p=0.045) | 0.98 (0.92-1.06, p=0.641) |
| **Colon** |  |  |  |
| White |  |  |  |
| Black | 1.14 (1.12-1.16, p<0.001) | 1.12 (1.10-1.15, p<0.001) | 1.06 (1.03-1.09, p<0.001) |
| Hispanic | 0.88 (0.86-0.91, p<0.001) | 0.85 (0.82-0.88, p<0.001) | 0.74 (0.71-0.78, p<0.001) |
| Asian | 0.79 (0.76-0.82, p<0.001) | 0.78 (0.74-0.81, p<0.001) | 0.74 (0.70-0.78, p<0.001) |
| Other | 0.94 (0.90-0.98, p=0.004) | 0.94 (0.89-0.99, p=0.018) | 0.90 (0.83-0.96, p=0.003) |
| **Rectal** |  |  |  |
| White | - | - | - |
| Black | 1.15 (1.10-1.21, p<0.001) | 1.16 (1.09-1.22, p<0.001) | 1.18 (1.10-1.27, p<0.001) |
| Hispanic | 0.83 (0.76-0.90, p<0.001) | 0.75 (0.67-0.83, p<0.001) | 0.75 (0.65-0.87, p<0.001) |
| Asian | 0.75 (0.66-0.87, p<0.001) | 0.74 (0.64-0.86, p<0.001) | 0.68 (0.56-0.82, p<0.001) |
| Other | 0.93 (0.83-1.05, p=0.247) | 0.91 (0.79-1.04, p=0.170) | 0.92 (0.75-1.13, p=0.426) |
| **Breast** |  |  |  |
| White | - | - | - |
| Black | 1.25 (1.23-1.27, p<0.001) | 1.23 (1.21-1.26, p<0.001) | 1.26 (1.23-1.30, p<0.001) |
| Hispanic | 0.81 (0.78-0.83, p<0.001) | 0.78 (0.75-0.81, p<0.001) | 0.73 (0.70-0.77, p<0.001) |
| Asian | 0.71 (0.67-0.74, p<0.001) | 0.68 (0.64-0.71, p<0.001) | 0.66 (0.62-0.71, p<0.001) |
| Other | 0.95 (0.91-0.99, p=0.028) | 0.88 (0.83-0.92, p<0.001) | 0.89 (0.82-0.96, p=0.001) |
| **Lung** |  |  |  |
| White | - | - | - |
| Black | 1.05 (1.03-1.06, p<0.001) | 1.00 (0.99-1.02, p=0.555) | 0.95 (0.94-0.97, p<0.001) |
| Hispanic | 0.91 (0.88-0.93, p<0.001) | 0.89 (0.87-0.92, p<0.001) | 0.81 (0.78-0.84, p<0.001) |
| Asian | 0.82 (0.80-0.85, p<0.001) | 0.80 (0.77-0.83, p<0.001) | 0.70 (0.67-0.73, p<0.001) |
| Other | 1.02 (0.98-1.05, p=0.344) | 1.01 (0.98-1.05, p=0.508) | 0.95 (0.90-0.99, p=0.017) |

Supplementary Figure 1a Clinical presentation of American Joint Commission on Cancer (AJCC) T3 or T4 on Clinical Staging by race across non-metastatic cancers

Supplementary Figure 1b Clinical presentation of American Joint Commission on Cancer (AJCC) Nodal positive disease on Clinical Staging by race across non-metastatic cancers

Supplementary Figure 2 Overall survival by race of each cancers (A) Oesophagus (B) Stomach (C) Liver (D) Pancreas (E) Colon (F) Rectal (G) Breast (H) Lung
